# SF3A3 Drives Tumorigenesis in Endometrial Cancer by Enhancing c‐FOS Expression and Represents a Potential Therapeutic Target

**DOI:** 10.1002/advs.202504184

**Published:** 2025-07-01

**Authors:** Wei Yu, Yi Gu, Hengrui Zhang, Wunan Huang, Lei Sun, Liangjian Ma, Xiaolei Liang, Lexin Liu, Guiping Chen, Weihua Wang, Zhongkai Cao, Xue Li, Xiangjun Chen, Lidan Hu

**Affiliations:** ^1^ Department of Nephrology Children's Hospital Zhejiang University School of Medicine National Clinical Research Center for Child Health Hangzhou 310052 China; ^2^ The First Affiliated Hospital of Zhejiang Chinese Medical University Zhejiang Provincial Hospital of Traditional Chinese Medicine Hangzhou 310028 China; ^3^ Eye Institute of Shandong First Medical University State Key Laboratory Cultivation Base Shandong Provincial Key Laboratory of Ophthalmology Qingdao 266071 China; ^4^ The First Hospital of Lanzhou University Key Laboratory of Gynecologic Oncology Gansu Province Lanzhou Gansu 730030 China; ^5^ Shandong Provincial Key Laboratory of Animal Cell and Developmental Biology School of Life Sciences Shandong University Qingdao 266237 China; ^6^ Engineer Center of Pharmaceutical Technology Tsinghua University Beijing 10084 China; ^7^ Department of Big Data in Health Science School of Public Health and The Second Affiliated Hospital Zhejiang University School of Medicine Hangzhou Zhejiang China; ^8^ Centre for Computational Biology (CCB) Duke‐NUS Medical School 8 College Road Singapore Singapore

**Keywords:** SF3A3, PEITC, c‐FOS, endometrial cancer, tumor progression

## Abstract

Aberrant alternative splicing plays a crucial role in tumorigenesis. Here, splicing factor 3A subunit 3 (SF3A3) is   significantly upregulated in endometrial cancer (EC) tissues and associated with poor prognosis. Functionally, SF3A3 drives tumor progression by promoting cell proliferation, suppressing apoptosis, and enhancing cisplatin resistance in vitro and in vivo. Mechanistically, SF3A3 regulates alternative splicing of fos proto‐oncogene, AP‐1 transcription factor subunit (c‐FOS), an oncogene linked to chemoresistance, resulting in a ≈2‐fold increase in full‐length c‐FOS expression and activation of downstream anti‐apoptotic pathways. Notably, phenylethyl isothiocyanate (PEITC) as a direct inhibitor of SF3A3 through database screening and biophysical validation via surface plasmon resonance and mass spectrometry is identified. PEITC reduces c‐FOS expression and induces apoptosis in EC cells. Moreover, encapsulating PEITC in a hydrogel delivery system significantly enhances its therapeutic efficacy by enabling controlled release, reducing dosing frequency, and improving clinical applicability. The therapeutic potential of SF3A3 inhibition is further validated using patient‐derived tumor‐like cell clusters (PTCs), where PEITC and the c‐FOS inhibitor T‐5224 exhibit synergistic effects in suppressing EC. Collectively, our findings establish SF3A3 as a novel oncogenic regulator in EC and highlight PEITC, particularly in its hydrogel formulation, as a promising therapeutic strategy for improving clinical outcomes in EC patients.

## Graphical abstract

The role of splicing factor 3A subunit 3 (SF3A3) in driving endometrial cancer (EC) progression and potential therapeutic interventions. SF3A3, a core component of the U2 small nuclear ribonucleoprotein (U2 snRNP), is upregulated in endometrial cancer (EC) and promotes tumor growth and cisplatin resistance. RNA‐binding protein immunoprecipitation (RIP) pull‐down assays reveal that SF3A3 drives alternative splicing of fos proto‐oncogene, AP‐1 transcription factor subunit (c‐FOS), leading to sustained c‐FOS expression, increased B‐cell lymphoma‐2 (Bcl2)/Bcl‐2 associated protein (Bax) ratio, and reduced apoptosis. Targeting SF3A3 with phenylethyl isothiocyanate (PEITC) effectively suppresses tumor progression, induces apoptosis, and reduces c‐FOS expression in vitro, in vivo, and patient‐derived tumor‐like cell clusters (PTCs). Furthermore, hydrogel‐based PEITC delivery significantly enhances therapeutic efficacy, offering a promising, clinically feasible strategy for precision‐targeted treatment in EC.

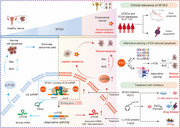

## 1 Introduction

Endometrial cancer (EC) is one of the most common gynecologic malignancies, with a rising global burden. Approximately 320000 new cases and 76000 deaths are reported worldwide each year^[^ ^1^ ^]^ Despite advancements in surgical interventions and chemotherapeutic strategies,^[^ ^2^, ^3^ ^]^ a significant proportion of EC patients experience recurrence and develop resistance to standard treatments, leading to poor clinical outcomes.^[^ ^4^, ^5^, ^6^, ^7^, ^8^ ^]^ Identifying novel molecular drivers contributing to EC progression and chemoresistance is essential to improving therapeutic strategies and patient prognosis.

Alternative splicing (AS) is a strictly regulated process that removes noncoding introns and joins coding exons in different combinations, greatly expanding transcriptome and proteome diversity.^[^ ^9^, ^10^, ^11^ ^]^ While AS is crucial for normal cellular function, its dysregulation has been implicated in cancer, promoting enhanced proliferation, evasion of apoptosis, and therapeutic resistance.^[^ ^12^, ^13^ ^]^ Increasing evidence suggests that AS abnormalities are particularly prevalent in EC, where splicing factor imbalances promote oncogenesis. For example, CircRAPGEF5 and splicing factor 3B subunit 1 (SF3B1) respectively promote EC progression by modulating AS of downstream genes in EC.^[^ ^14^, ^15^ ^]^ However, the specific role of AS in EC pathogenesis remains incompletely understood, particularly regarding the oncogenic functions of individual splicing factors.

The spliceosome, a multi‐subunit complex composed of small nuclear RNAs (snRNAs) and associated proteins, is central to AS process.^[^ ^16^, ^17^, ^18^ ^]^ Among its components, splicing factor 3A subunit 3 (SF3A3) is an essential member of the U2 small nuclear ribonucleoprotein (U2 snRNP), contributing to spliceosome assembly and pre‐mRNA recognition.^[^ ^18^ ^]^ Dysregulation of SF3A3 has been implicated in multiple cancers, including breast, bladder, and lung cancer, yet its role in EC remains unexplored.^[^ ^19^, ^20^, ^21^ ^]^ Given its critical function in mRNA maturation, SF3A3 may act as a key oncogenic regulator in EC by altering the splicing landscape of cancer‐associated genes.

Herein, we demonstrate that SF3A3 is frequently overexpressed in EC tissues and accelerates EC progression and cisplatin resistance. Mechanistically, SF3A3 regulates apoptosis and contributes to drug resistance, at least partially by modulating the AS of fos proto‐oncogene, AP‐1 transcription factor subunit (c‐FOS), a proto‐oncogene involved in cellular proliferation, drug resistance, and tumor growth. These findings identify SF3A3 as a promising target for novel therapeutic strategies in EC. Additionally, we evaluated the therapeutic efficacy of phenylethyl isothiocyanate (PEITC), a small molecule that directly inhibits SF3A3. Furthermore, encapsulating PEITC in a hydrogel delivery system significantly enhanced its therapeutic efficacy by enabling sustained release, reducing dosing frequency, and improving clinical feasibility. The cultivation conditions and drug concentrations determined in the PTCs model from EC patients support the potential for clinical translation. Our findings reveal a novel role of SF3A3 in EC progression and cisplatin resistance, and highlight PEITC, particularly in hydrogel formulation, as a promising strategy for improving treatment outcomes in EC patients.

## 2 Results

### 2.1 SF3A3 is Aberrantly Upregulated in EC Tissues and Promotes Poor Prognosis in Patients with EC

**Figure 1:**
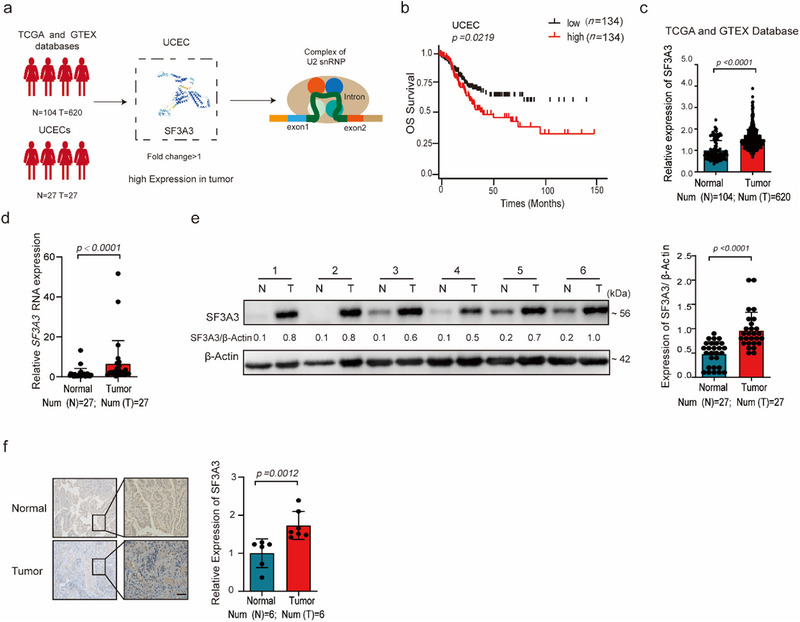
SF3A3 is frequently overexpressed in EC tumors and correlates with poor disease prognosis in EC patients. a) Schematic representation of the screening process for variable splicing factor SF3A3 in EC and its impact on alternative splicing. b) Kaplan–Meier curves illustrating overall survival (OS) predictions based on PSI values. Data were obtained from the UCSC Xena (https://xenabrowser.net/); *p < 0.05*, as compared with the low group; (*n* = 134 samples each group). c) SF3A3 is differentially overexpressed in EC tissues compared to normal tissues, based on TCGA and GTEx databases (https://gtexportal.org/ and https://cancergenome.nih.gov/); *p < 0.05*, as compared with the Normal group; (Normal = 104, tumor = 620 samples). d) *SF3A3* RNA expression is significantly upregulated in EC tissues (*n* = 27 samples each group). *p < 0.05*, as compared with the Normal group. e) SF3A3 protein expression is significantly elevated in EC tissues (*n* = 27 samples each group). *p < 0.05*, as compared with the Normal group. f) Immunohistochemistry analysis of SF3A3 expression in human endometrial and EC specimens (*n* = 6 samples each group). *p < 0.05*, as compared with the Normal group; Scale bars = 50 µm. Data are presented as mean ± S.D. from at least three independent experiments.

SF3A3 has been previously identified as a potential driver of tumor progression^[^ ^19^, ^20^, ^21^, ^22^ ^]^ (**Figure** **1**a). To elucidate its role, we analyzed SF3A3 expression abnormalities in EC tissues. Higher SF3A3 expression levels were strongly correlated with poor overall survival in UCEC patients, as shown by Kaplan–Meier survival analysis (Figure 1b). Further investigation using TCGA and GTEx databases confirmed significant upregulation of SF3A3 in EC tissues compared to normal samples (Figure 1c). Notably, SF3A3 expression varied slightly across tumor stages (Extended Data Figure 1a). High SF3A3 expression was most prominent in the POLE (ultramutated) and copy‐number high (serous‐like) subtypes, while lower expression was observed in the MSI (hypermutated) and copy‐number low (endometrioid) subtypes (Extended Data Figure 1a). Validation with our independent clinical samples dataset further confirmed that SF3A3 expression was significantly upregulated in EC tissues compared to tumor‐adjacent non‐cancerous endometrial tissues (Figure 1d–f and Extended Data Figure 1b–c; Table S7, Supporting Information). Collectively, these data indicated that elevated SF3A3 expression is strongly associated with worse clinical outcomes in EC patients and suggested that it may serve as a key biomarker for EC.

### 2.2 SF3A3 Promotes Proliferation and Xenograft Tumor Growth in EC

**Figure 2:**
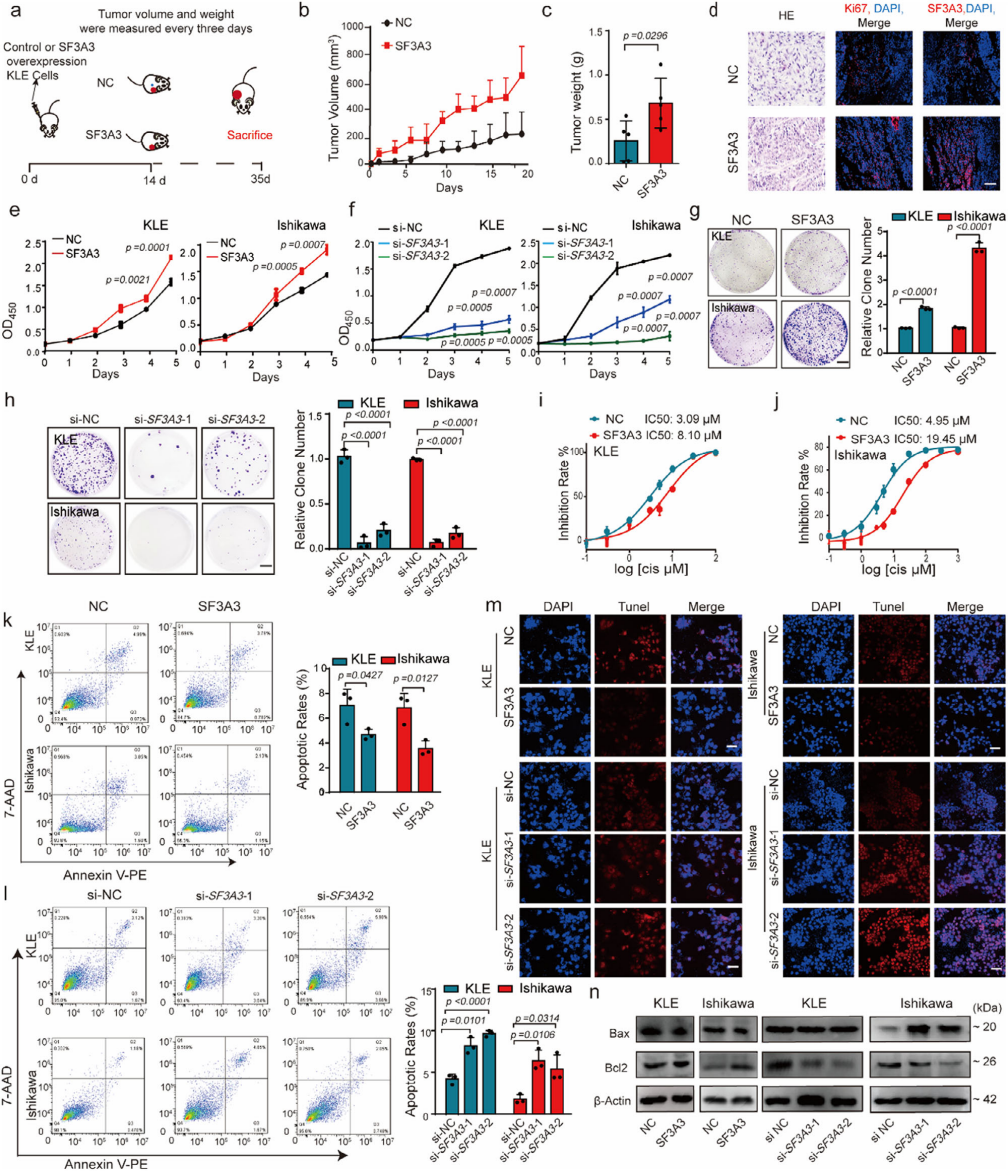
SF3A3 increases the oncogenic potential of EC cells. a) Schematic of the experimental protocol. Xenograft experiments by subcutaneous injection were conducted in KLE cells with vector or SF3A3 overexpression vector. On day 14, tumor volumes and mice weight were monitored; Animals were sacrificed on day 35, and tumor samples were collected for analysis. b–d) (b) Tumor volumes over time. (c) Tumor weights at endpoint. *p <* 0.05, as compared with the NC group; (*n* = 5 samples each group). (d) Representative images of SF3A3 and Ki67 immunofluorescence and HE staining in sections of excised tumors. Scale bars = 50 µm. e–h) Cell proliferation assays (e,f) and colony formation assays (g,h) were performed in SF3A3‐overexpressing or si‐*SF3A3* KLE and Ishikawa cells. *p <* 0.05, as compared with the NC or si‐NC group; (*n* = 3 independent experiments). i,j) Changes in cisplatin sensitivity following SF3A3 overexpression in KLE and Ishikawa cells. (*n* = 3 independent experiments). k,l) Apoptotic cells were detected via flow cytometry using Annexin V‐PE/7‐AAD staining in SF3A3‐overexpressing or si‐*SF3A3* KLE and Ishikawa cells. Data represent the percentage of early and late apoptotic cells *p <* 0.05, as compared with the NC or si‐NC group; (*n* = 3 independent experiments). m) Apoptotic cells detected by TUNEL staining in SF3A3‐overexpressing or si‐*SF3A3* KLE and Ishikawa cells. Scale bars = 20 µm. *p <* 0.05, as compared with the NC or si‐NC group; (*n* = 5 samples each group). n) Immunoblotting analysis of apoptotic markers (Bcl2 and Bax) in KLE and Ishikawa cells following SF3A3 overexpression or si‐*SF3A3*. *p <* 0.05, as compared with the NC or si‐NC group; (*n* = 3 independent experiments). Data are presented as mean ± S.D. from at least three independent experiments.

To investigate the functional role of SF3A3 in EC, we assessed its expression in the regular endometrial cell line (hEEC) and three EC cell lines. SF3A3 expression was markedly higher in EC cell lines, particularly KLE and Ishikawa cells, which were selected for further analysis (Extended Data **Figure** **2**a–c). We then modulated SF3A3 expression using lentiviral overexpression systems (pLV‐Puro‐SF3A3) in KLE and Ishikawa cells (Extended Data Figure 2d–h). In a mouse xenograft model, SF3A3‐overexpressing KLE cells were injected subcutaneously into nude mice. Tumor growth was monitored from day 14 when tumors reached ∼100 mm^3^. Overexpression of SF3A3 significantly increased tumor volume and weight after 35 days, without affecting the mice's body weight (Figure 2a–c, Extended Data Figure 2i,j). Immunofluorescence analysis revealed marked overexpression of SF3A3 and the proliferation marker Ki67 in tumors derived from SF3A3‐overexpressing cells compared to controls (Figure 2d and Extended Data Figure 2k). Consistent with in vivo findings, SF3A3 overexpression enhanced cell viability and colony formation in both KLE and Ishikawa cells after 10 days (Figure 2e,g). Conversely, silencing SF3A3 significantly inhibited cell viability and colony formation in both cell lines, further supporting its pro‐tumorigenic role (Figure 2f,h).

Furthermore, after overexpression of SF3A3, the IC_50_ of cisplatin in KLE cells changed from 3.09 to 8.10 µ_M_, and in Ishikawa cells, it changed from 4.95 to 19.45 µm (Figure 2i,j). Apoptosis assays, including TUNEL staining, Annexin V‐PE/7‐AAD staining, and flow cytometry, demonstrated that SF3A3 overexpression significantly reduced apoptosis in KLE and Ishikawa cells (Figure 2k,m, and Extended Data Figure 2l). Conversely, SF3A3 knockdown induced apoptosis in EC cells (Figure 2l,m and Extended Data Figure 2m). Immunoblotting analysis further revealed that SF3A3 overexpression increased the anti‐apoptotic Bcl2/Bax ratio, while knockdown reduced it (Figure 2n and Extended Data Figure 2n,o). Together, these findings demonstrate that SF3A3 drives tumor progression in EC by promoting cell proliferation, enhanced cisplatin resistance, and reduced apoptosis in vitro and in vivo.

### 2.3 The Genome‐Wide Landscape of SF3A3‐Binding Sites on RNA

**Figure 3:**
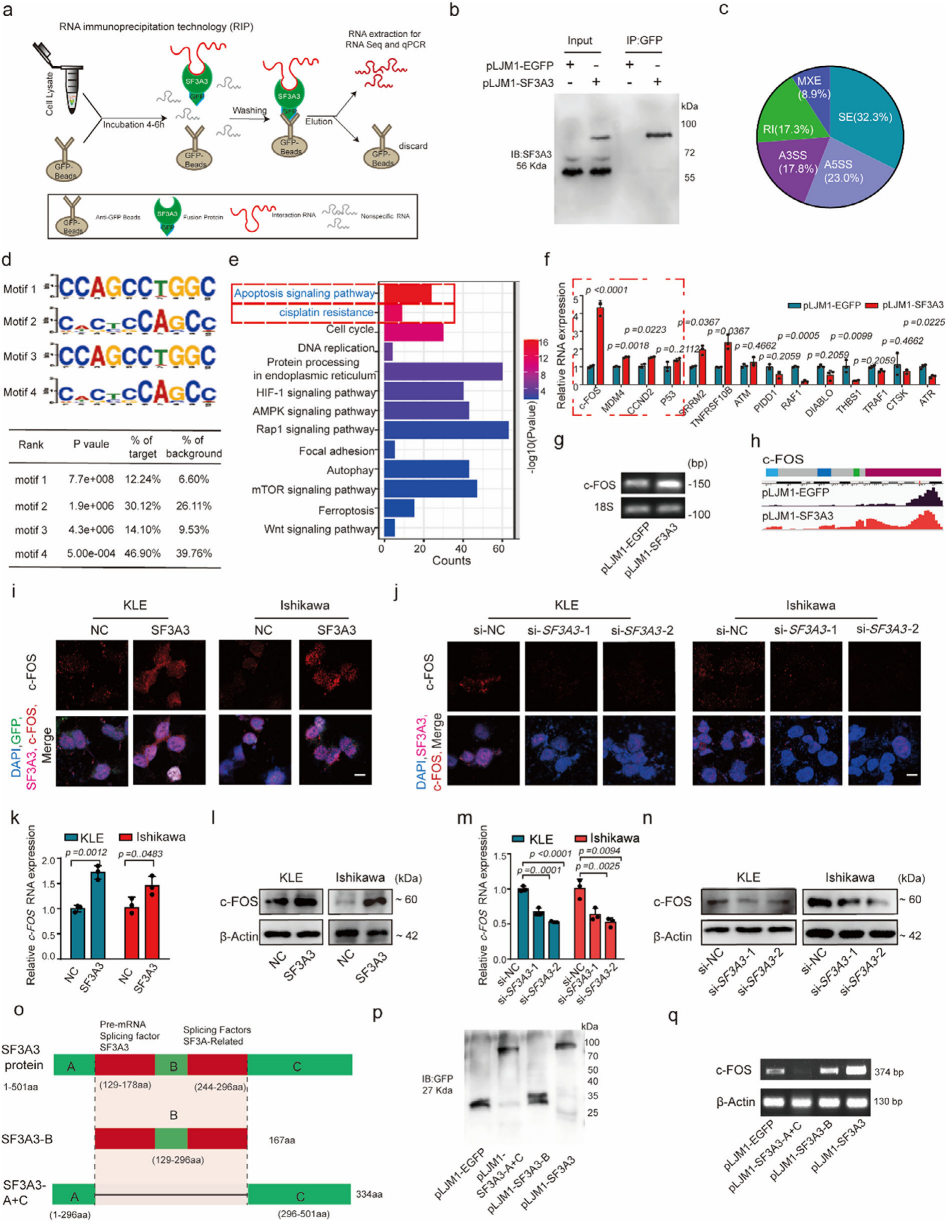
Identification of the genome‐wide SF3A3‐binding sites on RNA. a) Genome‐wide distribution of RNA targets identified by RNA Binding Protein Immunoprecipitation (RIP) followed by sequencing (RIP‐seq). (*n* = 3 samples each group). b) Protein validation of SF3A3 RIP pulldown. (*n* = 3 independent experiment). c) Alternative splicing (AS) events identified in RIP‐seq, classified into five categories: skipped exons (SE), retained introns (RI), mutually exclusive exons (MXE), alternative 5′ splice sites (A5SS), and alternative 3′ splice sites (A3SS). d) De novo motif analysis of SF3A3 RIP clusters, showing the top four SF3A3‐binding motifs ranked by HOMER cluster values. Sequence elements enriched in the top motifs are shown. e) KEGG pathway enrichment analysis of SF3A3‐interacting genes in KLE cells. f) RT‐qPCR validation of the top four SF3A3‐binding motifs identified by RIP. *p <* 0.05, as compared with the pLJM1‐EGFP group; (*n* = 3 independent experiments). g) Semi‐quantitative RT‐qPCR validation of SF3A3‐regulated genes, *c‐FOS*. (*n* = 3 independent experiments). h) Schematic of the alternative splicing (AS) pattern and SF3A3‐binding sites in c‐FOS. Light blue, green, and red regions highlight the AS region and SF3A3‐binding sites. i,j) Hybridization chain reaction (HCR) analysis showing co‐localization of SF3A3 and c‐FOS in SF3A3‐overexpressing or si‐*SF3A3* KLE and Ishikawa cells. Overexpression of SF3A3 alters c‐FOS gene expression. Scale bar = 10 µm. *p < 0.05*, as compared with the NC or si‐NC group; (*n* = 5 samples each group). k,m) RT‐qPCR analysis of SF3A3‐regulated c‐FOS expression in KLE and Ishikawa cells. *p < 0.05*, as compared with the NC or si‐NC group; (*n* = 3 independent experiments). l,n) Immunoblotting analysis of SF3A3‐mediated changes in c‐FOS protein levels in KLE and Ishikawa cells. *p < 0.05*, as compared with the NC or si‐NC group; (*n* = 3 independent experiments). o) Pattern diagram of constructing variable splicing mutant plasmids. p) Immunoblotting analysis of transfecting variable splicing mutant plasmids (*n* = 3 independent experiments). q) RT‐PCR assays validating SF3A3‐mediated alternative splicing in c‐FOS via transfecting with variable splicing mutant SF3A3 plasmids (*n* = 3 independent experiments). Data are presented as mean ± S.D. from at least three independent experiments.

As a splicing factor, SF3A3 may influence cancer progression through alternative splicing (AS) regulation. To identify its RNA targets, we performed RNA‐binding protein immunoprecipitation (RIP) followed by high‐throughput RNA sequencing (RNA‐seq) in KLE cells (**Figure** **3**a,b). RIP is a technique for studying the binding of intracellular RNA to proteins. Compared to control cells, RIP‐Seq results revealed that 2451 AS events enriched in SF3A3‐expression cells (Figure 3c–e, Extended Data Figure 3a). These events were categorized into five AS types: skipped exons (SE), retained introns (RI), mutually exclusive exons (MXE), alternative 5′ splice sites (A5SS), and alternative 3′ splice sites (A3SS). SE and RI represented the most abundant categories (Figure 3c).

To characterize the interaction of SF3A3 with RNA, we used the HOMER algorithm to identify RNA motifs recognized by SF3A3 (Figure 3d). Gene Ontology (GO) and Kyoto Encyclopedia of Genes and Genomes (KEGG) enrichment analysis of SF3A3‐modulated AS targets revealed significant involvement in apoptosis, cisplatin resistance, cell cycle regulation, focal adhesion, and DNA repair pathways (Figure 3e). To validate the RIP‐seq results, we selected four representative alternative splicing (AS) targets—*c‐FOS, CCND2, MDM4*, and *P53*—based on their percent spliced‐in (PSI) values and false discovery rates (FDRs) between SF3A3‐overexpressing and control cells. RT‐qPCR confirmed that SF3A3 regulated all four targets, with c‐FOS showing the most prominent changes (Figure 3f). Among them, c‐FOS, a core component of the activator protein‐1 (AP‐1) complex, has been closely associated with tumor progression, chemoresistance, and apoptosis, which supported its selection for in‐depth investigation.^[^ ^23^, ^24^, ^25^ ^]^

Semi‐quantitative PCR further validated these results, showing that SF3A3 overexpression significantly increased c‐FOS transcript levels (Figure 3g). Motif analysis revealed two high‐scoring SF3A3‐binding motifs near the exon 2–3 junctions, along with two additional motifs located within exonic regions (Figure 3h). Further studies showed that SF3A3 promoted the retention of introns 2 and 3, but not intron 1, and primarily facilitated the production of full‐length *c‐FOS* mRNA spanning exons 1–4, whereas intron‐containing isoforms were rarely detected (Extended Data Figure 3b). Integration of RIP‐seq peak mapping and RT‐PCR data suggested that SF3A3 bound directly to the exon 2–3 region of c‐FOS pre‐mRNA.

This binding interaction was further supported by hybridization chain reaction (HCR) analysis, which showed enhanced fluorescence intensity and co‐localization of SF3A3 with c‐FOS pre‐mRNA in SF3A3‐overexpressing cells (Figure 3i and Extended Data Figure 3c,d), with reciprocal signal reduction observed in SF3A3 knockdown cells (Figure 3j and Extended Data Figure 3e,f). RT‐qPCR and immunoblotting analysis confirmed that SF3A3 overexpression elevated both RNA and protein levels of c‐FOS, increasing protein levels by 1.55‐fold in KLE cells and 2.13‐fold in Ishikawa cells (Figure 3k,l and Extended Data Figure 3g). Conversely, SF3A3 knockdown significantly reduced c‐FOS expression (Figure 3m,n and Extended Data Figure 3h). To determine the functional domains of SF3A3 involved in c‐FOS regulation, we examined its protein structure. We identified the 129–178 amino acid (aa) region as the pre‐RNA binding domain and the 244–296 aa region as the splicing regulatory domain, based on InterPro annotations (https://www.ebi.ac.uk/interpro/protein/UniProt/Q12874/) (Figure 3o). Truncation of the 129–296 aa impaired SF3A3's regulatory activity, which resulted in reduced *c‐FOS* mRNA (Figure 3p,q), indicating the importance of this region in modulating c‐FOS expression.

To further expand the understanding of SF3A3's oncogenic network, we investigated additional targets identified by RIP‐seq. Notably, CCND2 and MDM4, both involved in cell cycle progression, were also validated as SF3A3‐regulated genes. Overexpression of SF3A3 elevated both RNA and protein levels of CCND2 and MDM4 (Extended Data Figure 3i–m). These findings suggested that SF3A3 promoted endometrial cancer progression through the modulation of c‐FOS and by coordinating a broader oncogenic splicing network.

### 2.4 High SF3A3 Expression Induces Cisplatin Resistance and Oncogenic Activity via c‐FOS Regulation

**Figure 4:**
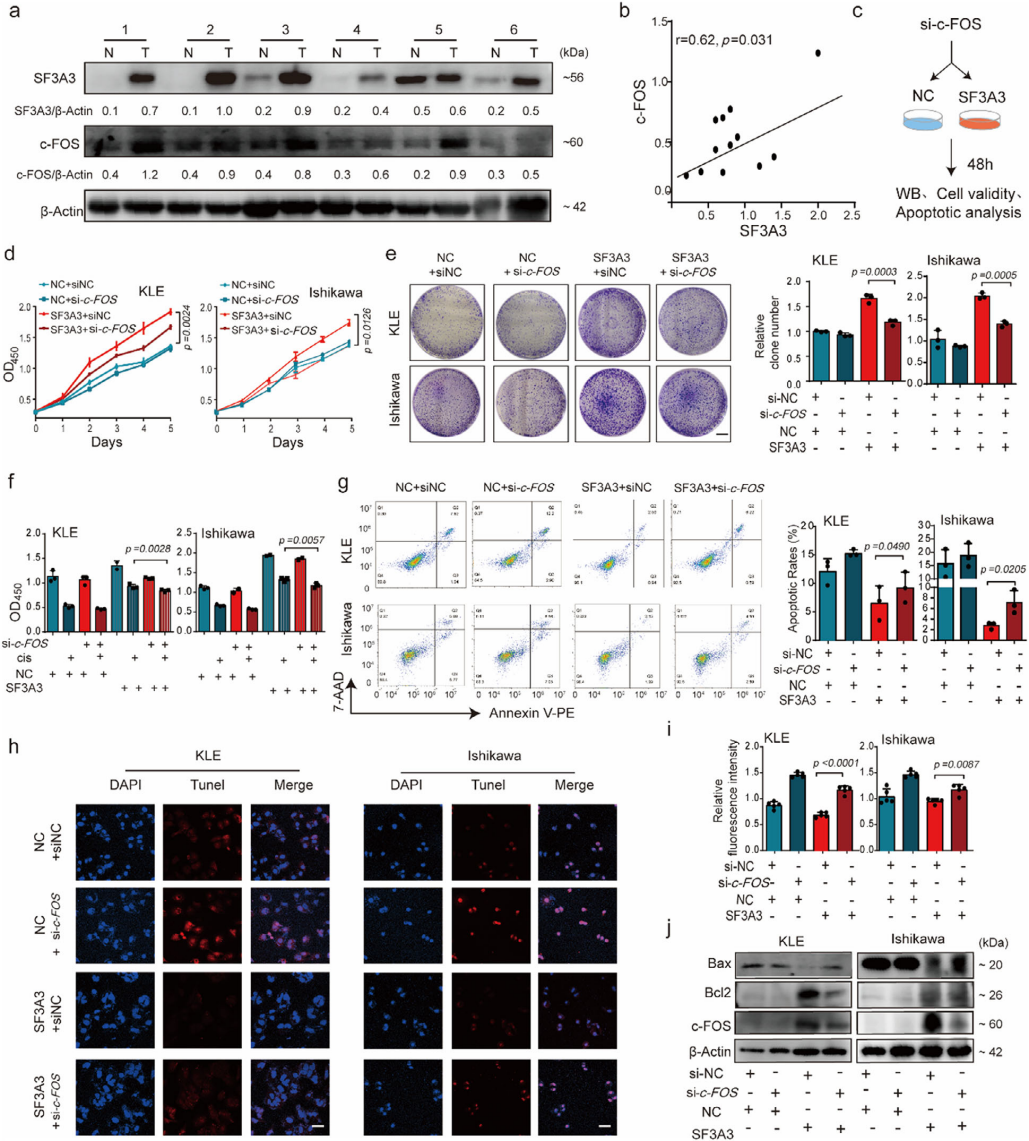
The oncogenic roles of SF3A3 in EC are mediated through c‐FOS expression. a) Immunoblotting analysis of c‐FOS and SF3A3 protein levels in paired adjacent noncancerous endometrium tissues (N) and human EC tissues. (*n* = 6 samples each group). b) Correlation analysis of protein SF3A3 and c‐FOS using paired adjacent noncancerous endometrium tissues (N) and human EC tissues (*n* = 6 samples each group). c) Functional analysis of the relationship between SF3A3 and c‐FOS in EC cells. Following SF3A3 overexpression, siRNA‐mediated knockdown of *c‐FOS* was used to evaluate cell apoptosis, proliferation, and cisplatin resistance: d) Growth curves in KLE and Ishikawa cells. *p <* 0.05, SF3A3+si‐*c‐FOS* group as compared with the SF3A3+si‐NC group; (*n* = 3 independent experiments). e) colony formation assays in KLE and Ishikawa cells. *p <* 0.05, SF3A3+si‐*c‐FOS* group as compared with the SF3A3+si‐NC group; (*n* = 3 independent experiments). f) Changes in cisplatin sensitivity following SF3A3 overexpression in KLE and Ishikawa cells. *p <* 0.05, SF3A3+si‐*c‐FOS* group as compared with the SF3A3+si‐NC group; (*n* = 3 independent experiments). g) Percentage of apoptotic cells analyzed by flow cytometry. *p <* 0.05, SF3A3+si‐*c‐FOS* group as compared with the SF3A3+si‐NC group; (*n* = 3 independent experiments). h,i) Percentage of apoptotic cells analyzed by TUNEL. Scale bars = 20 µm. *p <* 0.05, SF3A3+si‐*c‐FOS* group as compared with the SF3A3+si‐NC group; (*n* = 5 samples each group). j) Immunoblotting analysis of c‐FOS, Bcl2, and Bax protein levels to investigate whether c‐FOS can rescue the loss of SF3A3 function in terms of apoptosis and proliferation. *p <* 0.05, SF3A3+si‐*c‐FOS* group as compared with the SF3A3+si‐NC group; (*n* = 3 independent experiments). Data are presented as mean ± S.D. from at least three independent experiments.

Analysis of cell proliferation in vitro and EC growth in vivo strongly indicated that SF3A3 and c‐FOS have tumor‐suppressive effects. To further investigate the pathological significance of our findings, we examined SF3A3 and c‐FOS expression in 6 paired human EC lesions and adjacent noncancerous tissue samples. The results showed that SF3A3 and c‐FOS protein levels were significantly increased and Bcl2 was slightly increased in EC lesions compared to adjacent noncancerous tissue (**Figure** **4**a) and showed a moderate correlation between the expression of SF3A3 and c‐FOS (Figure 4b). Similar trends were observed in immunohistochemistry (IHC) results of clinical EC tissues and adjacent normal samples and mouse xenograft tumors (Extended Data Figure 4a,c).

To determine whether SF3A3's oncogenic effects rely on c‐FOS regulation, we performed rescue experiments. c‐FOS knockdown counteracted cell growth and apoptosis induced by SF3A3 overexpression (Figure 4c). Additionally, c‐FOS knockdown reversed SF3A3‐induced proliferation and clonogenic activity (Figure 4d,e). Importantly, cisplatin resistance and apoptosis mediated by SF3A3 overexpression were reduced when c‐FOS was silenced (Figure 4f–i). Immunoblotting analysis revealed that c‐FOS knockdown significantly decreased Bcl2 levels and increased Bax levels in SF3A3‐overexpressing cells (Figure 4j and Extended Data Figure 4d,e). These findings revealed a previously uncharacterized SF3A3–c‐FOS axis that drove EC progression and chemoresistance.

### 2.5 PEITC Anti‐Tumor Effects are Mediated by SF3A3 Inhibition

**Figure 5:**
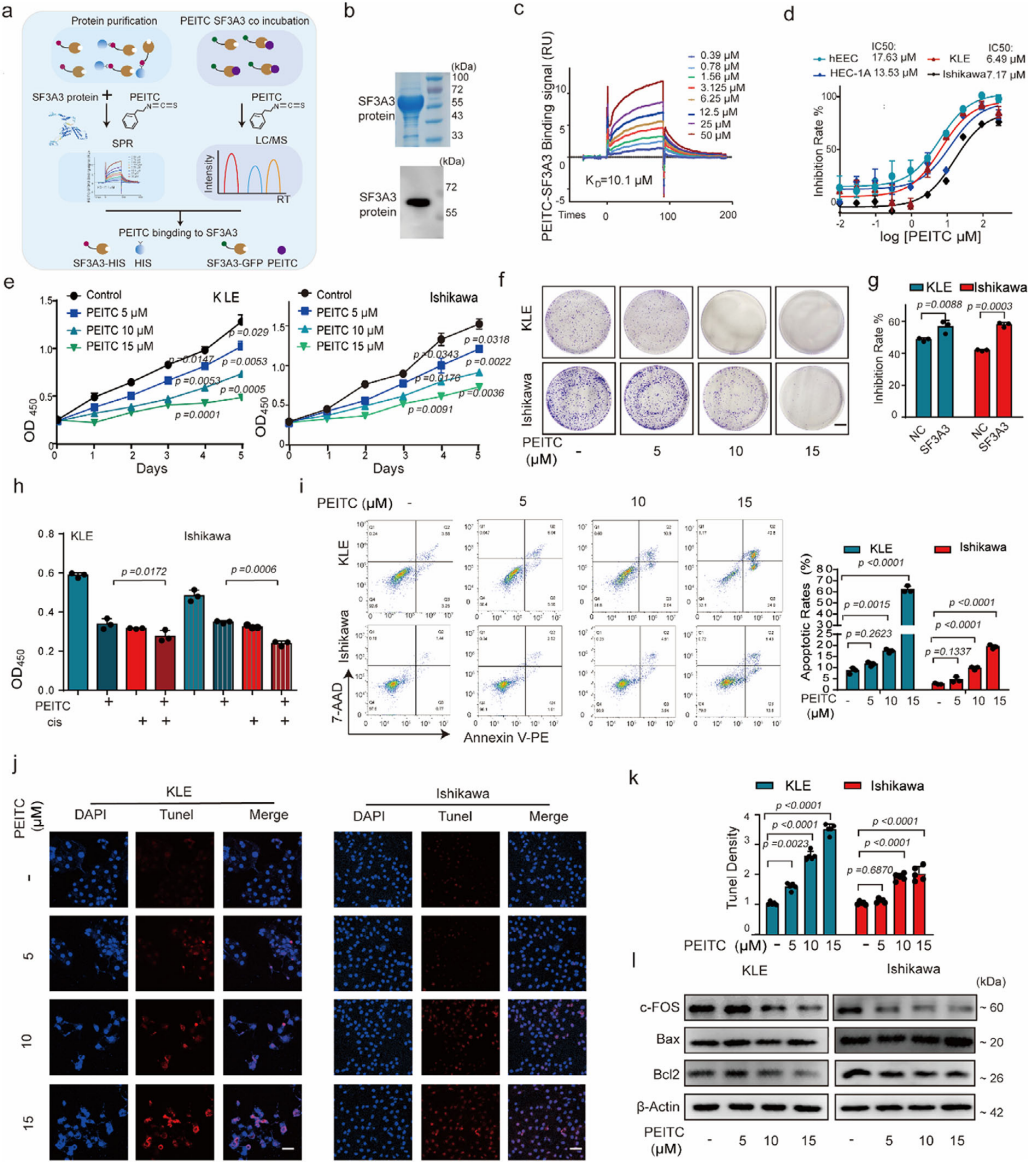
PEITC inhibits EC cell proliferation by targeting SF3A3 and cell apoptosis. a) Schematic of the experimental protocol. verification of the binding between PEITC and SF3A3 via SRP and mass spectrometry. b) Coomassie brilliant staining and immunoblotting of SF3A3 protein. c) Surface plasmon resonance (SPR) analysis demonstrating the binding interaction between SF3A3 protein and the small molecule PEITC. K_D_ = 10.1 µm. d) Inhibitory effects of PEITC on human EC cell proliferation, measured using a CCK‐8 assay. (*n* = 3 independent experiments). e) Quantification of KLE and Ishikawa cell viability over multiple days of PEITC treatment, as assessed by a CCK‐8 assay. *p < 0.05*, as compared with the Control group; (*n* = 3 independent experiments). f) Colony formation assays showing the effects of PEITC treatment on KLE and Ishikawa cells cultured for 10 days. *p < 0.05*, as compared with the control group; (*n* = 3 independent experiments). g) The change of PEITC on EC proliferation with SF3A3 overexpression (PEITC 10 µm). *p < 0.05*, SF3A3 group as compared with the NC group; (*n* = 3 independent experiments). h) Changes in cisplatin efficacy after combined treatment with cisplatin (3 µm) and PEITC (10 µm) for 48 hours. *p < 0.05*, PEITC+ cis group as compared with the cis group; (*n* = 3 independent experiments). i) Flow cytometry analysis of apoptosis in KLE and Ishikawa cells treated with PEITC, stained with Annexin V‐PE/7‐AAD to measure apoptosis, necrosis, and cell death. *p < 0.05*, as compared with the Control group; (*n* = 3 independent experiments). j,k) TUNEL staining of KLE and Ishikawa cells following PEITC treatment. Scale bars = 20 µm. *p < 0.05*, as compared with the Control group; (*n* = 5 samples each group). **l,** Immunoblot analysis of c‐FOS, BAX, and Bcl2 expression levels in KLE and Ishikawa cells after PEITC treatment. *p < 0.05*, as compared with the Control group; (*n* = 3 independent experiments). Data are presented as mean ± S.D. from at least three independent experiments.

Given the oncogenic role of SF3A3 in EC, we sought to identify a small‐molecule inhibitor of SF3A3. Our previous research suggests that PEITC may be a potential inhibitor of SF3A3^[^ ^22^ ^]^. Further verification of the binding between PEITC and SF3A3 was conducted via Surface plasmon resonance (SPR) and mass spectrometry (**Figure** **5**a). SPR experiments confirmed a strong binding interaction between SF3A3 and PEITC, with a K_D_ value of 10.1 µm (Figure 5b,c). Mass spectrometry further revealed that SF3A3‐overexpressing samples exhibited significantly higher PEITC content than controls (Extended Data Figure 5a,b).

The anti‐tumor activity of PEITC was evaluated in normal endometrial cells and EC cell lines, revealing that cells with high SF3A3 expression (KLE and Ishikawa) were more sensitive to PEITC, as indicated by lower IC_50_ values (Figure 5d). PEITC treatment induced a dose‐ and time‐dependent decrease in cell viability and colony formation in KLE and Ishikawa cells (Figure 5e,f and Extended Data Figure 5c). Cells overexpressing SF3A3 exhibited enhanced sensitivity to PEITC (Figure 5g and Extended Data Figure 5d). Moreover, the combination of PEITC and cisplatin effectively reduced cisplatin resistance induced by SF3A3 overexpression (Figure 5h). PEITC treatment significantly increased apoptosis in KLE and Ishikawa cells, as demonstrated by Annexin V‐PE/7‐AAD staining, TUNEL assays, and reduced expression of c‐FOS and the anti‐apoptotic protein Bcl2, accompanied by an increased Bcl2/Bax ratio (Figure 5i–l and Extended Data Figure 5 e,f). These results indicated that the anti‐EC effects of PEITC were mediated, at least in partly, by SF3A3 inhibition.

### 2.6 PEITC shows Tumor Suppression in Xenograft Tumor Growth and Patient Derived Tumor Like Cell Clusters (PTCs) in EC

**Figure 6:**
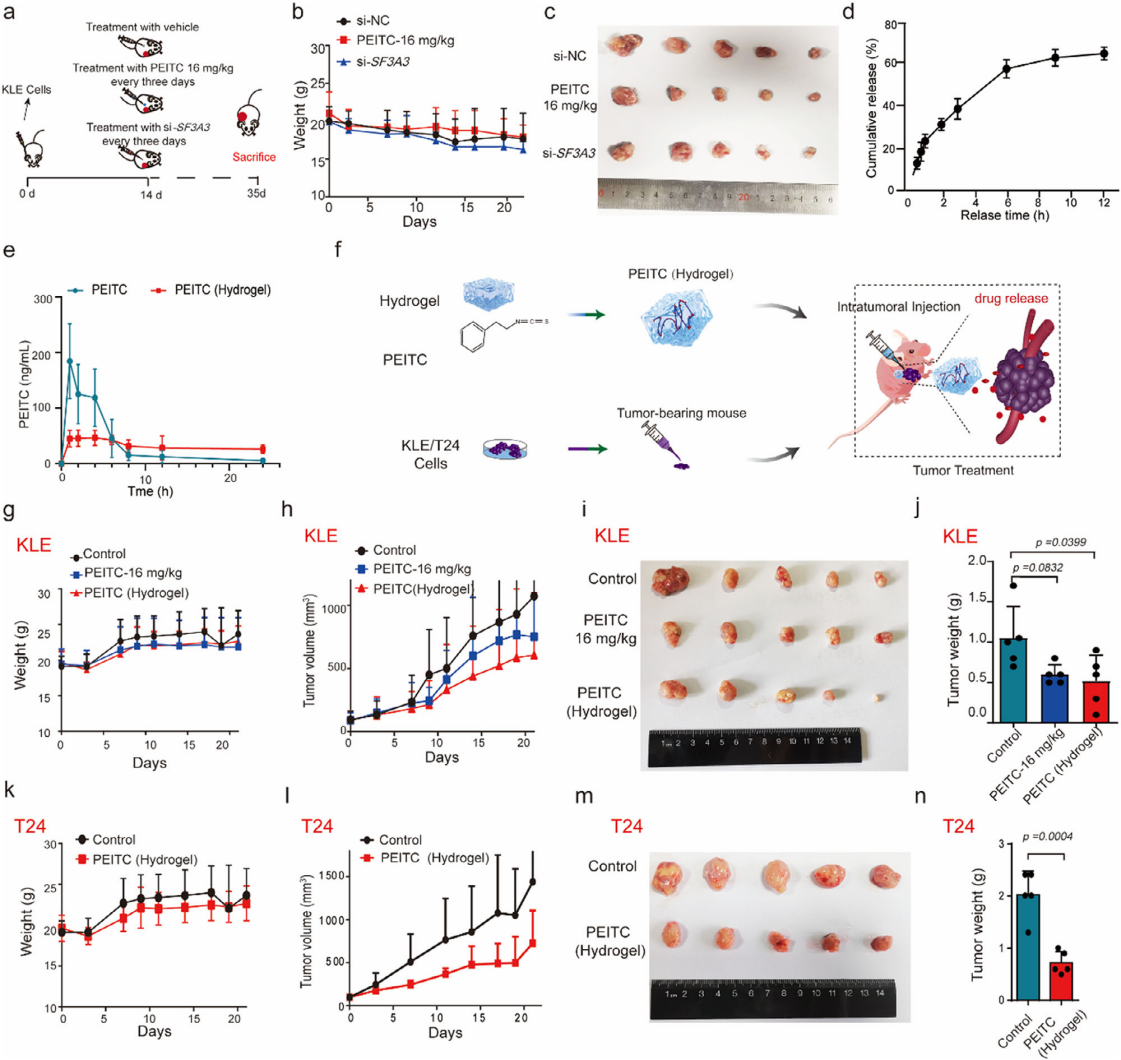
Effect of SF3A3 and PEITC on EC xenograft growth in mice in EC samples. a) Experimental protocol. Groups of athymic BALB/c nude mice were injected subcutaneously with 10^6^ KLE cells. After the tumor volume reaches 100 mm, 5 nm si‐*SF3A3*, si‐NC or 16 mg kg^−1^ PEITC was injected every 3 days via the intratumoral injection or intragastric administration route. On day 35, animals were sacrificed and tumors samples were collected for analysis. (*n* = 5 samples each group). b) Body weight change. c) Tumor photographs. d) % PEITC release from the hydrogel versus time (h) at 37 °C. e) Plasma concentration versus time profiles of PEITC following single administration. Red color‐PEITC‐loaded hydrogel (Hypo Hypodermic injection) green color‐Free PEITC (oral). (n = 6 samples each group). f) Schematic representation of the anti‐tumor mechanism of PEITC encapsulated in hydrogel. (*n* = 5 samples each group). Once tumors reached ∼100 mm^3^, mice were treated with PEITC encapsulated in hydrogel (administered once weekly) or PEITC via oral gavage (every 3 days). g–n) (g,k) Body weight changes in mice during the experiment. (h,l) Tumor volumes measured at designated time points. (i,m) Photographs of excised tumor xenografts. (j,n) Tumor weights at endpoint. *p < 0.05*, as compared with the Control group; (*n* = 5 samples each group).

To confirm whether PEITC's in vitro anti‐tumor effects translated in vivo, we used a mouse xenograft model. Nude mice were subcutaneously injected with KLE cells, and when tumors reached ∼100 mm^3^, the mice were treated with si‐*SF3A3* or PEITC administered orally every 3 days until day 35. Tumor load analysis showed that PEITC and siRNA significantly reduced tumor volume and weight without affecting body weight (**Figure** **6**a–c, Extended Data Figure 6a,b). Immunofluorescence assays revealed decreased expression of c‐FOS and the proliferation marker Ki67 in PEITC‐treated tumors compared to controls (Extended Data Figure 6c). These findings confirm that PEITC effectively suppresses tumor growth in vivo by targeting SF3A3.

To improve the therapeutic efficacy of PEITC, we encapsulated it in a hydrogel delivery system. Hydrogel‐based systems offer an effective platform for drug delivery due to their high water content, excellent biocompatibility, minimal cytotoxicity, and superior drug encapsulation capacity.^[^ ^26^ ^]^ Drug metabolism revealed almost 65% of PEITC was released within 12 h (Figure 6d). In vivo pharmacokinetic analysis and biodistribution studies demonstrated that hydrogel‐encapsulated PEITC (PEITC‐Hydrogel) resulted in more stable plasma concentrations (Figure 6e) and lower PEITC accumulation in major organs such as the liver and kidneys at 2 and 4 h post‐injection, compared to free PEITC formulations (Extended Data Figure 6e). These results demonstrate the feasibility of the PEITC‐Hydrogel for the sustained release of PEITC at EC tumor tissue.

Using xenograft models with KLE cells (EC) and T24 cells (bladder cancer), we compared the effects of hydrogel‐encapsulated PEITC (PEITC‐Hydrogel) with oral administration. PEITC‐Hydrogel demonstrated superior tumor suppression in both xenograft models, significantly reducing tumor volume and weight with no observed difference (Figure 6f–n, Extended Data Figure 6a–d). Over time, the in situ hydrogels underwent gradual biodegradation and were fully resorbed within 14 days, accompanied by a mild inflammatory response (Extended Data Figure 6f), which was similar to previous report.^[^ ^26^ ^]^ Toxicological assessments (16 mg kg^−1^ PEITC‐Hydrogel) further confirmed that the PEITC‐Hydrogel group exhibited reduced hepatorenal toxicity (Extended Data Figure 6g,h). Collectively, these findings underscore the therapeutic potential of PEITC‐loaded hydrogels to enhance anti‐tumor efficacy while reducing systemic toxicity and dosing frequency.

**Figure 7:**
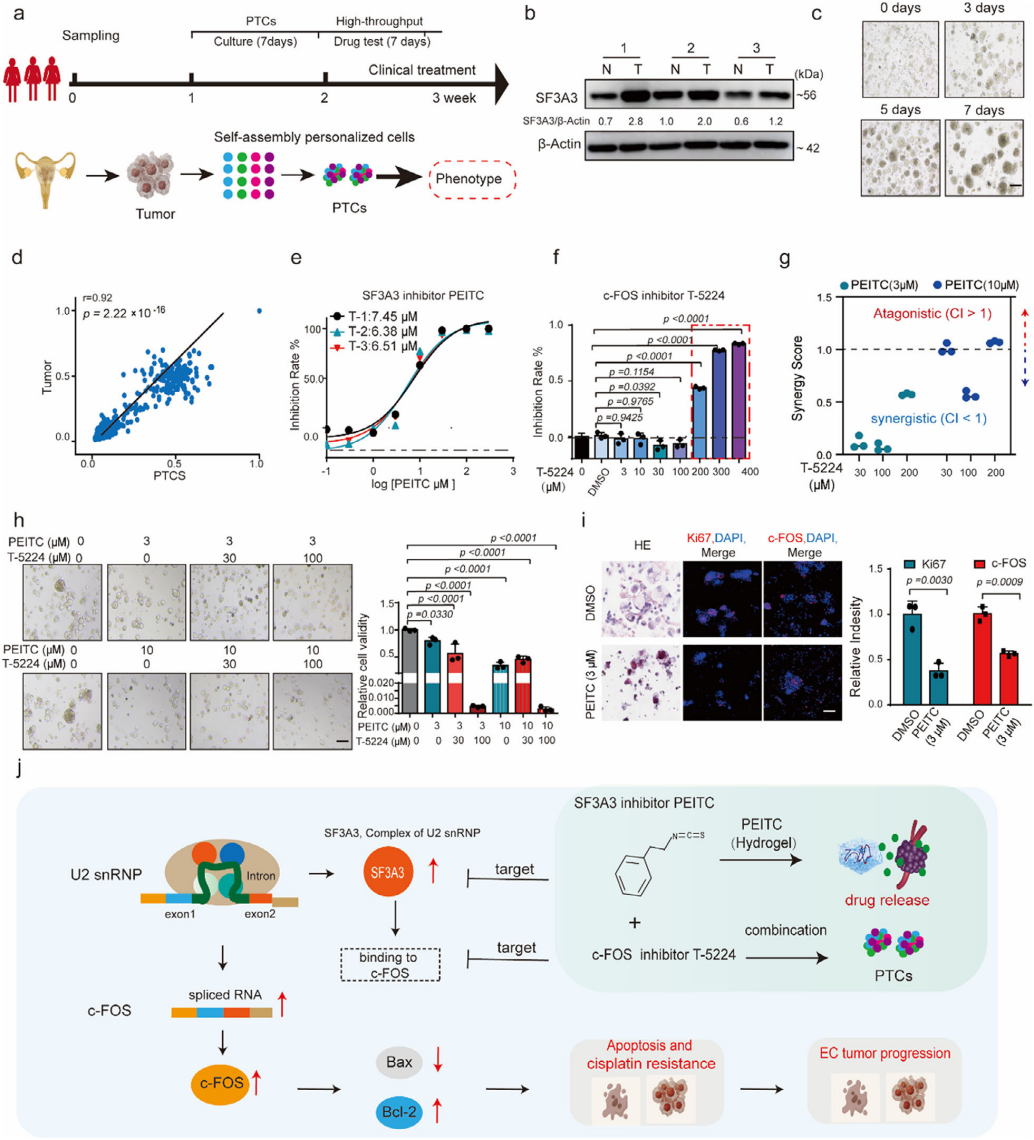
Effect of PEITC on PTCs constructed in EC samples. a) PTCs construction and drug treatment mode diagram. b) Immunoblotting analysis of SF3A3 in EC tissues. *p* < 0.05, as compared with the Control group; (*n* = 3 independent experiments). (*n* = 3 samples each group). c) Images from different periods during the PTCs construction process. Scale bars = 50 µm. (*n* = 3 samples each group). d) Scatter plots display the correlation between the DNA variant allele frequency (VAF) of EC organoids and their parental tumors. The figures show the linear regression line (black) and the corresponding r and P values. e,f) The therapeutic concentration gradient of drug c‐FOS inhibitor T‐5224 and PEITC in PTCs. *p < 0.05*, T‐5224 (200300400 µm) group as compared with the DMSO group; (*n* = 3 samples each group). g) Drug synergy plots for PEITC and the c‐FOS inhibitor‐T‐5224. Each data point indicates an average synergy score from a single dose–response matrix experiment performed. The Chou Talalay method was used to calculate the synergistic effect of drugs: the effects of drugs were divided into three parts: the effects of PEITC and T‐5224 drugs alone (E1 and E2) and the synergistic effect of PEITC and T‐5224 drugs (E1+E2); Calculate Composite Index (CI): Use the formula CI = (E1+E2) / (E1 * E2) to evaluate synergistic effects. If the CI is less than 1, it indicates that the combination of drugs has a synergistic effect; If CI is equal to 1, it means there is no synergistic effect; If CI is greater than 1, it indicates an antagonistic effect. h) Images of PEITC and T‐5224 treatment for 48 h in PTCs from EC patient. Scale bar = 50 µm. *p < 0.05*, as compared with the (PEITC+T‐5224‐0 µM) group; (*n* = 3 samples each group). i) Representative IHC images showing Ki67, c‐FOS expression and HE staining after PITEC treatment in PTCs from EC patient. *p* < 0.05, as compared with the Control group; (*n* = 3 samples each group). Scale bars = 50 µm. Data are presented as mean ± S.D. from at least three independent experiments. j) Schematic diagram illustrating the mechanism by which SF3A3 influences EC progression through c‐FOS regulation. Data are presented as mean ± S.D. from at least three independent experiments.

To better explore the prospects of PEITC in clinical treatment, we constructed PTCs, and the expression of SF3A3 showed higher in EC tumor tissues than normal tissues^26^ (**Figure** **7**a–c, Extended Data Figure 7a). To assess the genomic fidelity of PTCs, we isolated genomic DNA from both PTC cells and matched primary tumor tissues and performed whole exome sequencing (WES). The results demonstrated a high degree of genomic concordance, supporting the use of PTCs as a reliable preclinical model for downstream drug screening applications (Figure 7d, Extended Data Figure 7b,c). Interestingly, PEITC showed significant efficacy with a low concentration and c‐FOS inhibitor T‐5224 inhibited the survival rate of PTCs at high concentrations (Figure 7e,f). Then, the average cell area was measured at different PEITC and T‐5224 concentrations, and it was found that PEITC and T‐5224 inhibited cell size in a dose‐dependent manner (Figure 7e,f). We first plotted relative inhibition rates as a function of PEITC concentration with increasing T‐5224 dosage (Extended Data Figure 7d). We next compared the synergy summary scores from the different matrix sizes via Chou Talalay method (Figure 7g, Extended Data Figure 7d). The combination treatment of PEITC and T‐5224 showed significant synergistic effects at PEITC 3 µM in combination with T‐5224 30, 100, or 200 µm (Figure 7g,h). Immunofluorescence assays revealed decreased expression of c‐FOS and the proliferation marker Ki67 in PEITC‐treated PTCs compared to controls (Figure 7i). The above research indicates that there is a significant synergistic effect between SF3A3 inhibitors and downstream c‐FOS inhibitors, significantly increasing the drug use scenarios and efficacy of PEITC in EC. These findings suggest that targeting SF3A3–c‐FOS signaling with PEITC and T‐5224 may provide a promising therapeutic strategy for EC patients (Figure 7j).

## 3 Discussion

Endometrial cancer (EC) remains a significant clinical challenge, with a rising global incidence and limited effective treatments for advanced or recurrent cases. Although alternative splicing (AS) has emerged as a critical regulatory mechanism in cancer, its role in EC progression and chemoresistance remains poorly understood.^[^ ^18^, ^27^ ^]^ In this study, we identify SF3A3 as a key oncogenic splicing factor in EC and demonstrate that its overexpression drives tumor growth, enhances cisplatin resistance, and suppresses apoptosis. Mechanistically, SF3A3 promotes the alternative splicing of c‐FOS, a proto‐oncogene implicated in cancer progression, leading to increased full‐length c‐FOS expression and upregulation of the anti‐apoptotic Bcl2/Bax ratio. Furthermore, we identify PEITC as a direct SF3A3 inhibitor, which effectively suppresses tumor growth and enhances cisplatin sensitivity. Importantly, the combination of PEITC and the c‐FOS inhibitor T‐5224 demonstrated synergistic effects in patient‐derived tumor‐like cell clusters (PTCs). Our findings position SF3A3 as a novel therapeutic target and establish a clinically relevant strategy for improving EC treatment.

Dysregulation of AS is a hallmark of cancer, enabling tumor cells to evade apoptosis, enhance proliferation, and develop drug resistance.^[^ ^9^, ^11^, ^27^, ^28^ ^]^ In EC, AS has been increasingly recognized as a key contributor to tumor progression; however, the specific mechanisms and functional consequences of these aberrant splicing events remain poorly defined.^[^ ^29^, ^30^ ^]^ Several core splicing factors—such as SF3B1, SRSF2, and U2AF1—have been implicated in the pathogenesis of both hematologic and solid tumors by altering the splicing of critical oncogenes and tumor suppressors. Among these, SF3B1, a well‐characterized component of the U2 snRNP complex, has been reported to recognize 3′ splice sites and promote EC progression by regulating KSR2 mRNA maturation.^[^ ^30^, ^31^ ^]^ SF3B1 mutations are also frequently observed in uveal melanoma, with hotspot mutations detected in ≈20% of cases.^[^ ^31^ ^]^

In contrast, SF3A3, another core member of the U2 snRNP complex, has remained largely underexplored in EC. Most existing studies on SF3A3 have focused on its aberrant expression across various cancer types, but its functional role and mechanistic contributions to tumorigenesis—particularly in EC—have not been previously characterized. Our study fills this important gap by identifying SF3A3 as a novel oncogenic regulator in EC, functioning through direct regulation of c‐FOS alternative splicing, which promotes tumor proliferation and chemoresistance. Notably, recent studies have begun to reveal broader oncogenic functions of SF3A3 in other cancers. For example, SF3A3 was shown to synergize with YTHDF2 to drive hepatocellular carcinoma progression,^[^ ^32^ ^]^ and to selectively regulate MYC‐driven splicing and metabolic reprogramming in breast cancer, where SF3A3 levels modulate MYC‐induced plasticity and oncogenesis.^[^ ^21^ ^]^ In non‐small cell lung cancer, circSCAP can interact with SF3A3 to suppress malignancy, and in bladder cancer, E2F6/KDM5C promotes SF3A3 expression through a hypomethylated promoter region.^[^ ^19^, ^20^ ^]^ More recently, structural studies by Shi et al. revealed that the separator helix of SF3A3 inserts into the gap between pre‐mRNA and U2 snRNA, stabilizing the pre‐mRNA–snRNA complex and supporting its core splicing function.^[^ ^18^, ^33^ ^]^ Together, these findings, along with our work in EC, highlight SF3A3 not only as a structural component of the spliceosome, but also as a dynamic regulator of cancer‐specific splicing programs, with distinct oncogenic effects depending on tissue context. Our study is the first to demonstrate the direct functional role of SF3A3 in EC progression, thus providing novel mechanistic insight and laying the groundwork for future translational exploration.

Through RIP‐seq, we identified c‐FOS, a proto‐oncogene and core component of the AP‐1 complex, as a direct target of SF3A3‐mediated alternative splicing.^[^ ^34^ ^]^ SF3A3 promoted the inclusion of exons 2 and 3 in c‐FOS pre‐mRNA, generating the full‐length isoform that activates the Bcl2/Bax signaling pathway, thereby enhancing tumor survival and cisplatin resistance.^[^ ^23^, ^24^, ^34^ ^]^ Quantitatively, SF3A3 overexpression led to a 1–2‐fold increase in c‐FOS protein, a 1–2 fold increase in the Bcl2/Bax ratio, and a 2–3‐fold increase in cisplatin IC_50_, consistent with enhanced chemoresistance. In contrast, SF3A3 knockdown reversed these effects, sensitizing cells to cisplatin.^[^ ^35^ ^]^ These findings demonstrate that SF3A3 exerts its oncogenic and chemoprotective effects primarily through c‐FOS regulation, positioning SF3A3 as a key driver of endometrial cancer progression and a potential therapeutic target to overcome chemotherapy resistance. c‐FOS knockdown confirmed that SF3A3 exerted its oncogenic effects primarily through c‐FOS regulation, further solidifying its role as a key driver of EC progression.

Given the oncogenic role of SF3A3 in EC, we sought to identify a small‐molecule inhibitor capable of targeting SF3A3‐driven tumorigenesis. Using bioinformatics screening and biophysical validation, we identify PEITC as a direct SF3A3 inhibitor. PEITC has established anti‐tumor activity in lung, breast, bladder, and colorectal cancers.^[^ ^36^, ^37^, ^38^, ^39^, ^40^ ^]^ Surface plasmon resonance (SPR) and mass spectrometry analysis confirmed a strong binding interaction between PEITC and SF3A3. Importantly, PEITC restored cisplatin sensitivity in SF3A3‐overexpressing EC cells, underscoring its potential as a dual‐function therapy that both inhibited tumor growth and reversed drug resistance. Pharmacokinetic studies show that PEITC has high oral bioavailability, ranging from 90% to 114% at doses of 10–100 µm kg^−1^ in rats.^[^ ^40^, ^41^, ^42^ ^]^ However, frequent dosing and low solubility limit its clinical utility. To address these challenges, we developed a hydrogel‐based delivery system for sustained PEITC release. Hydrogel has a porous 3D network structure and can be used as the carrier for controlled‐release extracellular vesicle (EVs) to enable the sustained release of drug at injury sites.^[^ ^43^, ^44^ ^]^ In this study, we used a hydrogel to encapsulate PEITC, enabling site‐specific delivery, reduced dosing frequency, and improved bioavailability. Pharmacokinetic and biodistribution studies demonstrated stable plasma PEITC concentrations and reduced hepatic and renal accumulation compared to free PEITC, thereby minimizing off‐target toxicity. Moreover, in vivo xenograft models using both KLE (endometrial cancer) and T24 (bladder cancer) cells confirmed that PEITC‐hydrogel achieved superior tumor suppression with reduced systemic toxicity.

To further validate the therapeutic potential of SF3A3 inhibition, we employed a patient‐derived tumor‐like cell cluster (PTC) model, which recapitulates patient tumor heterogeneity and drug response.^[^ ^44^, ^45^ ^]^ PEITC exhibited strong anti‐tumor activity in PTCs, particularly when combined with the c‐FOS inhibitor T‐5224, demonstrating synergistic effects in reducing tumor cell viability. This combination strategy offers a clinically relevant approach to improve treatment efficacy and overcome resistance in EC patients.^[^ ^45^ ^]^ These results collectively demonstrate that PEITC's anti‐tumor efficacy is not solely due to general cytotoxicity, but is driven by its specific inhibition of the SF3A3–c‐FOS oncogenic axis. Our hydrogel‐based approach provides a clinically translatable strategy for targeted delivery, enhanced safety, and improved therapeutic index of SF3A3 inhibitors such as PEITC.

While our study establishes SF3A3 as a key oncogenic driver in EC and identifies PEITC as a promising therapeutic inhibitor, several limitations should be acknowledged. First, we primarily focused on the SF3A3–c‐FOS axis, yet it is likely that other SF3A3‐regulated splicing events also contribute to EC progression. Comprehensive transcriptome‐wide analysis are needed to uncover additional downstream targets and delineate their functional roles. Second, although patient‐derived tumor‐like cell clusters (PTCs) offer a robust and physiologically relevant model, the number of PTC samples used in this study was limited due to stringent tissue quality requirements and extended culture timelines. This limitation may constrain the generalizability of our findings and warrants validation in larger patient cohorts. Third, while PEITC has entered phase II clinical trials (NCT00691132), its anti‐tumor mechanisms are multifaceted. In addition to its direct binding to SF3A3, PEITC has been shown to modulate other signaling pathways, including HSF1 (heat shock transcription factor 1) and Nrf2 (nuclear factor erythroid 2‐related factor 2),^[^ ^46^ ^]^ both of which play important roles in cellular stress responses. Although our binding studies support a direct interaction between PEITC and SF3A3, potential off‐target effects cannot be fully excluded. Further investigation into PEITC's selectivity, as well as combination strategies that enhance specificity and therapeutic efficacy, will be critical for advancing SF3A3‐targeted therapy into clinical application.

Moving forward, our findings suggest that targeting the SF3A3–c‐FOS axis may synergize with existing therapies, including immune checkpoint inhibitors and targeted agents. Optimizing PEITC‐based combination strategies and hydrogel‐mediated drug delivery will be crucial for advancing SF3A3‐targeted therapy toward clinical application. Beyond the scope of EC, SF3A3 dysregulation has been observed in multiple malignancies. These findings suggest that targeting SF3A3 may hold therapeutic value in other chemoresistant tumors, warranting further investigation. In summary, our study highlights SF3A3 as a potential biomarker and therapeutic target in EC. It provides a strong foundation for future research aimed at developing precision therapies for endometrial cancer.

## 4 Conclusion

This study identifies SF3A3 as a critical oncogenic driver in EC. Its overexpression promotes tumor growth, cisplatin resistance, and apoptosis suppression through regulation the alternative splicing of c‐FOS. We further validate PEITC as a potent SF3A3 inhibitor that effectively suppresses tumor progression and restores cisplatin sensitivity. Hydrogel‐based delivery of PEITC significantly enhances its therapeutic efficacy by improving release control and reducing dosing frequency. PTC models further confirm the clinical potential of this approach. Together, these findings provide a strong preclinical rationale for targeting SF3A3 as a novel therapeutic strategy and pave the way for future translational research in EC.

## 5 Experimental Section

### Human Tissue Samples

EC specimens were collected from April 2022 to February 2024 February in the First Hospital of Lanzhou University. The EC samples were obtained from patients with EC who had not undergone any previous surgery or chemotherapy. Fresh tissue samples were collected within 2 h of surgery and were sliced to 5 mm^3^ and respectively immersed in 100 µL of TRIzol reagent (Thermofisher, #15 596 018) and protein lysate containing phosphatase inhibitors and protease inhibitors. The tissue samples were stored at −80 °C. All patients provided informed consent, and ethical approval was granted by the Ethics Committee for Clinical Research (Drugs and Devices) of Lanzhou University First Hospital (LDYYLL2024‐142).

The inclusion criteria for clinical samples include histological diagnosis of endometrial cancer, age ≥ 18 years, sufficient tumor tissue for analysis, no prior chemotherapy or radiotherapy before tissue collection, and the ability to provide informed consent. The exclusion criteria mainly include other concurrent malignant tumors, insufficient quality or quantity of tissue samples, previous treatments that may affect tissue integrity (such as neoadjuvant therapy), and inability to provide informed consent.

### Cell Culture and Reagents

The HEC‐1A, hEEC, Ishikawa and KLE cell lines were purchased from the China Center of American Type Culture Collection (ATCC, Wuhan, China). HEC‐1A cells were maintained in McCoy's 5A, hEEC and Ishikawa cells were maintained in DMEM and KLE cells were maintained in DMEM/F12 medium containing 10% fetal bovine serum (FBS) at 37 °C in a humidified atmosphere with 5% CO_2_. PEITC was obtained from Merck and PEITC's 10 mM liquor configuration: PEITC was resuspended in 10% DMSO, 40% PEG300, 5% Tween‐80 and 45% phosphate‐buffered saline (PBS) for experiments. Dilute 10 mm PEITC with PBS to 5‐10‐15 µm for relevant experiments, and use a mixed solvent without PEITC with the same ratio as 15 µm for the control group The remaining reagents and antibodies are listed in the table (Tables S1 and S2, Supporting Information).

### Construction of EC Cell Lines

To achieve SF3A3 overexpression (OE), the aforementioned conditions were repeated with overexpression vectors (pLJM1‐ SF3A3 and pLJM1‐EGFP; Qizhen Lake, HangZhou, China). The viral infection diversity (MOI): Ishikawa = 50; KLE = 10; Next, the medium was exchanged for fresh DMEM or DMEM/F12 medium and the cells were cultured for an additional 24 h. Stable SF3A3 overexpression cell lines were selected by growth in a medium containing 10 µm puromycin. SF3A3 siRNA (si‐*S* *F3A3* ‐1: 5′–GACUGCUGCUUAUCAACAATT–3′ and si‐*SF3A3* ‐2: 5′–GCUGCUCGUGAUAUGCUGUTT–3′) and nontargeting siRNA (si‐NC) were purchased from Shanghai Gene Pharma Co., Ltd (Shanghai, China) (The relevant siRNA sequences are displayed in Table S3, Supporting Information). Efficient si‐*SF3A3* or overexpression of *SF3A3* was verified by RT‐qPCR and immunoblotting. The efficiency percentages in siRNA are: qPCR KLE: si‐*SF3A3*‐1: 64.8% si‐*SF3A3*‐2: 69.5%; Ishikawa: si‐*SF3A3*‐1: 67.5% si‐*SF3A3*‐2: 55.2%. Immunoblotting: KLE: si‐*SF3A3*‐1: 54.1% si‐*SF3A3*‐2: 70.3%; Ishikawa: si‐*SF3A3*‐1: 94,3% si‐*SF3A3*‐2: 88.0%. The efficiency percentages in overexpression are: RT‐qPCR: KLE: 455.7% Ishikawa: 741.6%; Immunoblotting: KLE: 228.4% Ishikawa: 482.4%.

### Colony‐Forming Assay

KLE and Ishikawa cells were seeded in 6‐well culture plates at 4000 cells wel^l−1^ and treated with PEITC (5‐10‐15 µm) for 10 days (wild type cells) or 14 days (si‐NC, si‐*SF3A3*‐1, si‐*SF3A3*‐2 or NC, SF3A3 cells). Stain with 0.5% crystal violet (Solarbio Life Science, Beijing, China) for 15 min, wash off excess staining solution with PBS three times, take photos, and analyze the number of cell clones using Image J.

### Colorimetric Cell Proliferation Assay

Cells were seeded in 96‐well plates at 2000 cells well^−1^ and incubated at 37 °C with 5% CO_2_. Cell proliferation was assessed using the Cell Counting Kit‐8 kit (CCK‐8; Beyotime, Jiangsu, China) according to the manufacturer's instructions. At 0, 24, 48, 72, 96, and 120 h, the medium was replaced with 100 µL of 10% CCK‐8 reagent in a serum‐free medium. Absorbance was measured after 1 h using a microplate reader (Molecular Devices, USA).

### Flow Cytometry

Flow cytometry analysis for cell apoptosis was performed using an Annexin V‐PE/7‐AAD Apoptosis Detection Kit (Yeasen Biotechnology, #40310ES20, Shanghai, China). 5 × 10^5^ cells were digested with ethylenediaminetetraacetic acid (EDTA)‐free trypsin (Macgene, CC035) for 3 min, collected by centrifugation, washed with ice‐cold phosphate‐buffered saline (PBS), and resuspended at a density of 5 × 10^5^ cells mL^−1^ with 100 µL 1 × binding buffer. Then 5 µL Annexin V‐PE and 10 µL 7‐AAD were added and incubated for 10 min in the dark. Finally, cells were incubated with an additional 400 µL 1 × binding buffer and analyzed within 20 min by CytoFLEX S (Beckman Coulter Life Science). At least 1 × 10^4^ cells were collected to determine the percentage of apoptotic cells.

Annexin V‐PE and 7‐AAD are used with specific excitation and emission wavelengths: Annexin V‐PE (Ex = 488 nm, Em = 578 nm) emits orange‐red fluorescence and is detected via the FL2 channel, while 7‐AAD (Ex = 546 nm, Em = 647 nm) emits red fluorescence and is detected via the FL3 channel. Data analysis is performed using software to generate a two‐color dot plot with PE intensity on the x‐axis and 7‐AAD intensity on the y‐axis. A total of 10 000 cells are collected per sample. In typical experiments, three cell subpopulations are identified: live cells (low background fluorescence), early apoptotic cells (strong orange‐red fluorescence from PE), and late apoptotic cells (dual staining with PE and 7‐AAD). Cellular abundances were compared across distinct subgroups and validated subset differences using gating strategies in flow cytometry scatter plots, visualized with FlowJo software.

### TUNEL Assays

For TUNEL assays, KLE and Ishikawa cells were seeded in 12‐well culture plates at 1 × 10^6^ cells well^−1^ and incubated at 37 °C with 5% CO_2_. Treated with PEITC (5‐10‐15 µm) for 2 days (wild type cells) or 2 days (si‐NC, si‐*SF3A3*‐1, si‐*SF3A3*‐2 or NC, SF3A3 cells) respectively. Fixed with 4% paraformaldehyde and the TUNEL Cell Apoptosis Detection Kit (Beyotime, C1098) was used according to the manufacturer's protocol. Cell nuclei were stained with DAPI. Confocal images of cells were sequentially acquired with a Nikon A1 Ti system. TUNEL positive cells are evaluated based on fluorescence intensity as a percentage of total cells.

### Immunoblotting

Si‐*SF3A3* or overexpression of SF3A3 KLE and Ishikawa cells and cells were incubated with PEITC for 48 h were processed for immunoblotting. Primary antibodies against SF3A3 (ab157194) and a secondary FITC‐conjugated goat anti‐rabbit IgG (H+L) antibody (AS024) were purchased from Abclonal Biotechnology (Abclonal, Wuhan, China). Horseradish peroxidase (HRP)‐conjugated anti‐mouse IgG (AS061) was purchased from Abclonal Biotechnology (Abclonal, Wuhan, China) and anti‐rabbit IgG secondary antibodies (4414S) were purchased from Cell Signaling Technology (Beverly, MA, USA) (1 h). GoldBand 3‐color Low Range Protein Marker (Yeasen Biotechnology, #20350es, Shanghai, China and Vazyme Biotech, #MP102‐01, Nanjing, China). Protein bands were visualized using enhanced chemiluminescence reagents (Millipore, Burlington, MA).

### RNA Binding Protein Immunoprecipitation Assay (RIP)‐seq

RIP is a technique for studying the binding of intracellular RNA to proteins. 10 million KLE (pLJM1‐EGFP or pLJM1‐SF3A3) cells were collected and lysed in 500 µL RIP buffer (20 mM Tris‐HCl pH 7.4, 100 mm KCl, 1.5 mm MgCl_2_, 0.2 mm EDTA, 1% NP‐40, 100U RNase inhibitor, 1 × cocktail). Whole‐cell extracts were incubated with 30 µg GFP‐beads (AlpalifeBio, # ktsm1301) overnight for 3 h at 4 °C. Immunocomplexes were washed with washing buffer for 3 times (20 mM Tris‐HCl pH 7.4, 100 mm KCl, 1.5 mm MgCl2, 0.2 mM EDTA), treated with proteinase K, and RNA was extracted using henol/chloroform/isoamyl alcohol (125:24:1). RNA library standard: Total RNA content>1 µg, concentration>20 µg µL^−1^; OD_260/280_: 1.7‐2.5; OD_260/230_: 0.5‐2.5, RIN>6; 28s/18s:1‐5. RNA Clean reads were aligned to the hg38 genome using bowtie2 (v2.5.1). Both input and RIP samples were prepared for next‐generation sequencing by Genomic consistency analysis conducted by Beijing Biomarker Technologies Co. Ltd. (Beijing, China). RIP seq raw data are uploaded to the GEO database, GEO Submission (GSE287381) [NCBI tracking system #24 990 495].

### RNA Sequencing Data Processing and Analysis

Raw sequencing data were processed using fastpv0.23.4 to filter out low‐quality and low‐complexity reads and remove adapter sequences.^[^ ^47^ ^]^ Cleaned reads were aligned to the hg38 reference genome in paired‐end mode using bowtie2 (v2.5.1).^[^ ^48^ ^]^ The resulting BAM files were converted to bigWig format using bam Coverage from deep tools (v3.5.3)^[^ ^49^ ^]^ for chromosome coverage visualization. Custom R scripts were utilized to generate chromosome coverage plots.

Metagene plots were created using compute Matrix and plot Profile functions from deep tools (v3.5.3). Peak calling was performed with MACS2 (v2.2.9.1)^[^ ^50^ ^]^ using the call peak function, and highly reproducible peaks across replicates were identified using IDR (v2.0.4.2).^[^ ^51^ ^]^ After quality control checks, data from replicates were merged to generate a combined set of peaks for each group. The overlap and intersection of peaks between cell lines were assessed using Venn diagrams generated by the intersect function in bed tools (v2.27.1).^[^ ^52^ ^]^ Motif analysis was conducted with MEME (v5.0.2),^[^ ^53^ ^]^ restricting motif length to 6–10 bp and retaining motifs with significant E‐values (< 10^−3^). Peak annotation, including overlapping gene locations, was performed with bed tools (v2.27.1) and reference gene annotation data from encode regions. Functional annotation of genes was done using the DAVID online database https://david.ncifcrf.gov/; last accessed October 2024), focusing on cell cycle, apoptosis regulation‐related genes. Genomic distribution of peaks was visualized using IGV (v2.16.2).^[^ ^54^ ^]^

rMATS (RNA‐MATE Analysis Tool for Splicing) is a bioinformatics tool designed specifically for RNA splicing difference analysis (http://rnaseg‐mats.sourceforge.net/index.html). There are five main splicing modes: exon skipping, mutually exclusive exons, alternative 5′splice site, alternative 3′ splice site, and intron retention. In terms of quantitative analysis, mats calculate the PSI value (percent spliced in) of each event based on transcript covered splice site reads and exon covered reads to quantify the proportion change of splicing isoforms. At the same time, the software uses nonparametric statistical tests (such as Wilcoxon rank sum test) to evaluate the significance of differences between conditions, and combines multiple hypothesis correction (such as Benjamini‐Hochberg method) to control the false discovery rate, so as to ensure the reliability of the results.

### Hybridization Chain Reaction (HCR)

On the first day, KLE and Ishikawa (pLJM1‐EGFP or pLJM1‐SF3A3) samples were fixed with 4% paraformaldehyde (Servicebio, G1101) and washed with PBST 5 times for 5 min. Hybridization buffer (50% formamide, 5 × sodium chloride sodium citrate, 9 mm citric acid pH = 6.0, 0.1% Tween‐20, 50 µg mL^−1^ heparin, 1 × Denhardt's solution, 10% dextran sulfate) and primers were added, primers were showed in Table S6 (Supporting Information) and samples were incubated at 37 °C overnight. Second day: washing with 300 µL probe wash buffer (50% formamide, 5 × sodium chloride sodium citrate, 9 mm citric acid pH = 6.0, 0.1% Tween‐20, 50 µg mL^−1^ heparin) 3 times for 15 min at 37 °C, followed by washing with 1 mL5 × sodium chloride sodium citrate and 0.1% Tween‐20 at room temperature 3 times for 15 min. Then amplification was performed by incubating samples with amplification buffer (5 × sodium chloride sodium citrate, 0.1% Tween‐20, 10% dextran sulfate) and amplifier (SangonBiotech, China) at room temperature overnight. Third day: washing with 1 mL 5 × SSCT (0.75 m NaCl, 0.075 m Sodium citrate, 0.1% Tween‐20) 5 min for 2 times, then washing with 1 mL 5 × SSCT 30 min for 2 times, then washing with 1 mL 5 × SSCT for 5 min. Then samples were imaged with a Nikon A1 Ti system. All assays were repeated at least three times.

### Protein Mass Spectrometry Detection of PEITC and SF3A3

10 million KLE (pLJM1‐EGFP or pLJM1‐SF3A3) cells were collected and lysed in 500 µL IP buffer (10 mm Tris‐HCl pH 7.5;150 mm NaCl;0.5 mm EDTA;0.1% SDS;1% Triton X‐100;1% Deoxycholate). Whole‐cell extracts were incubated with 30 µg GFP‐beads (AlpalifeBio, ktsm1301) for 3 h at 4 °C. Immunocomplexes were washed with washing buffer (50 mM Tris‐HCl pH 7.5;150 mm NaCl;1 mM EDTA) for 3 times (20 mM Tris‐HCl pH = 7.4, 100 mm KCl, 1.5 mm MgCl2, 0.2 mM EDTA), Then used for co‐incubation with PEITC (15 µM) respectively, followed by washing with washing buffer three times and validation using mass spectrometry experiments. Divided into a control group and an SF3A3 overexpression group.

MS data were acquired using electrospray ionization in positive ion mode, m/z was 186‐130.1. Other MS settings include: sheath gas temperature 250 °C, sheath gas flow 11 L/min, Nebulizer 20 psi, Capillary 3000 V, Nozzle voltage 1500 V, gas temperature 200 °C. Raw data were processed using QQQ Quantitative Analysis (Quant‐My‐Way) (Agilent) for peak detection, alignment and integration.

### Surface Plasmon Resonance

The binding of PEITC to purified human SF3A3 protein (General biol, Anhui, China) was measured by SPR using a Biacore 8K system (Cytiva, America), which is from the Analysis Center of Agrobiology and Environmental Sciences, Zhejiang University. Briefly, the SF3A3 protein was immobilized on a CM7 sensor chip (Cytiva) to a level of 30 520.7 response units (RUs) using Biacore (8K). For steady state affinity analysis, the PEITC was dissolved in 1 × PBS ‐P+ buffer containing 5% DMSO at concentrations of 0.39,0.78,1.56,3.125,6.25,12.5, 25 to 50 µm, and were run across the chip. Each sample that was bound to the surface was associated for 90s at a flow rate of 30 µL min^−1^. Dissociation of sensor chips was performed for 90s. The dissociation constant (KD) was fitted and recorded by Biacore Insight Evaluation Software (Cytiva) using the One‐to‐One analysis model. All assays were repeated at least three times.

### RNA Isolation, RT‐PCR, and RT‐qPCR

Total RNA was isolated from cultured cells using TRIzol reagent (Thermofisher, #15 596 018), and cDNA was generated using PrimeScript RT Master Mix (Vazyme Biotech, #R333‐01, Nanjing, China) according to the manufacturer's protocol. The cDNA product was subjected to quantitative real‐time PCR (qPCR) using SYBR Premix Ex‐Taq (Tli RNaseH Plus) (Vazyme Biotech, #Q121‐02/03, Nanjing, China). Expression levels of target genes were normalized to those of the housekeeping gene actin, and data are present as mean ± SD. For RT‐PCR assays, cDNAs were amplified by 2 × Hieff Robust PCR Master Mix (Yeasen Biotechnology, #10106ES08, Shanghai, China), and the PCR products were then separated by DNA gel electrophoresis. The primer sequences used for RT‐qPCR and RT‐PCR are listed in Tables S4 and S5 (Supporting Information), respectively. RT‐qPCR and RT‐PCR primers were synthesized in Hangzhou Qingke Biotechnology and sequences are described in Tables S4 and S5 (Supporting Information). All assays were repeated at least three times.

### PEITC(Hydrogel) Synthesis

The PEG‐PLGA‐PEG triblock copolymer was synthesized via ring‐opening polymerization. Briefly, PEG (Mn = 1500, 4 g) was dried under vacuum at 120 °C for 3 h to eliminate residual moisture. Subsequently, lactic acid (LA, 5 g), glycolic acid (GA, 0.5 g), and stannous octoate (0.05 g) were added as monomers and catalysts respectively. The mixture was stirred at 150 °C under an argon atmosphere for 24 h. The crude product was precipitated three times in deionized water, filtered, dialyzed, and lyophilized. Structural confirmation of the resulting copolymer was performed using ¹H NMR spectroscopy in CDCl₃ (Extended Data Figure 8a,b).

Preparation of PLGA‐PEG‐PLGA‐@PEITC gel: To prepare the drug‐loaded hydrogel, 1 g of the synthesized PLGA‐PEG‐PLGA copolymer was dissolved in 5 mL of deionized water and dispersed via ultrasonication to obtain a homogeneous precursor solution. PEITC was then incorporated into this solution at a final concentration of 20 mg mL^−1^. Upon warming to 37 °C, the mixture underwent a sol–gel transition, forming the injectable PLGA‐PEG‐PLGA@PEITC hydrogel.

The degradation of the PLGA‐PEG‐PLGA hydrogel is primarily governed by the hydrolysis of ester bonds within the PLGA segment. Water molecules cleave these bonds, progressively reducing the polymer into short‐chain oligomers of lactic acid and glycolic acid. Continued hydrolysis leads to the formation of monomeric units, which are metabolized into carbon dioxide and water, ultimately achieving complete biodegradation and mineralization.

### Mouse Xenograft Experiments

The mouse xenograft experiment obtained female thymus free BALB/c nude mice (5–6 weeks old, weight 18–22 g) from the Charles River (Beijing, China) group.

KLE cells (WT or NC or SF3A3 cells) or T24 cells were collected and resuspended in serum‐free medium at a concentration of 10^6^ cells mL^−1^ and subcutaneously injected into one side. After 14 days, the tumor volume reached≈100mm,^[^ ^3^ ^]^ and the animals were injected intraperitoneally with indicated doses of PEITC (16 mg/kg, every two days), PEITC(Hydrogel) (20 µL, 20 mg mL^−1^, once a week), si‐*SF3A3*‐1 (30 µL 5 nm, every two days) or vehicle (PBS or hydrogel). Every two days, all groups were used a vernier caliper to measure the length and width of the tumor and were used a balance to measure the weight of the mice. After three weeks of administration, euthanize the mice and collect tumor samples. Measure the tumor weight of the mice and divide them into two groups. One group is fixed in formalin for subsequent HE staining and immunofluorescence staining. HE staining is conducted by the Wuhan Servicebio Technology Co., Ltd (Wuhan, China). Another group is used for immunoblotting experiments; Tumor volume calculation formula: 1/2 * length * width * width; All animal experiments were conducted using double‐blind methods. All animal experiments were approved by the Ethical Review Committee of Laboratory Animal Welfare, Zhejiang University (ZJU20230106).

### Immunofluorescence (IF) Staining

Tumors were excised from the mice within 4 h of the final treatment dose and then fixed in 10% formalin for 24 h and stored in 70% ethanol in PBS at 4 °C. Tissues were sliced into 8 µm sections and stained with primary antibodies against SF3A3 (Cell Signaling Technology, #8057S) or Ki67 (Abclonal #A2094) followed by anti‐rabbit IgG secondary antibodies (Cell Signaling Technology, Beverly, MA, USA). Tissues were imaged using an inverted fluorescence microscope at 20 × magnification.

### Culture of PTCs from Solid Tumors

Studies on PTC were conducted by GeneX Precision Medicine Co., Ltd. (Zhejiang China). These EC specimens were collected at the first Hospital of Lanzhou University. Collected samples were conditioned in ice‐cold PBS with 10 mm HEPES and 100 U/mL penicillin‐streptomycin (Thermo Fisher Scientific). Tissues were washed with PBS at least five times. Necrotic areas and adipose tissue were removed as possible. Tissues were minced into small pieces and digested in 5 mL PBS/EDTA 1 mm containing collagenase l, ll, and IV (Thermo Fisher Scientific) 200U mL^−1^, each, at 37 °C for 1 h, the digestion was pipetted every 15 min to facilitate cell release. 40 µm filters were used to collect dissociated cells. After 10 min’ centrifugation (300 x g, 4 °C) cell pellets were resuspended in PTCs growth medium and seeded in low‐attachment‐surface dish at the concentration of 10^5^ cells cm^−2^. Cells were cultured in an incubator at 37 °C, 5% CO_2_. PTCs growth medium was refreshed every 2–3 days, as necessary.

### Cell Viability Assay for PTCs

For PTCs/spheres in a 96‐well plate, the medium was half removed and replaced with 80 µL Celltiter‐Gloreagent from a cell viability assay (Promega). Cells were incubated at room temperature for 1 h, and viability readings were obtained using a Synergy plate reader (BioTek).

### Genomic Consistency of PTCs with the Original Tumors

Genomic consistency analysis was carried out by Majorbio BioPharm Technology Co., Ltd. (Shanghai, China). Genomic DNA was extracted using the CTAB method, and its quality was assessed via 1% agarose gel electrophoresis to detect degradation or contamination. DNA concentration and purity were measured with a NanoDrop ND‐2000 spectrophotometer. Only high‐quality DNA samples (OD_260/280_ between 1.8 and 2.0; OD_260/230_ ≥ 2.0) were used for library preparation. Genomic DNA was randomly fragmented to 180–280 bp (Covaris, Massachusetts, USA). Fragment ends were blunted and A‐tailed before ligation with adapter oligonucleotides. Ligated fragments were enriched by PCR, indexed, and hybridized with biotin‐labeled probes. Exons were captured using streptavidin‐coated magnetic beads, further enriched by PCR, purified (Beckman Coulter, USA), quantified (Agilent Bioanalyzer 2100), and sequenced on the DNBSeq T7 platform.

Low‐quality reads (phred score < 20), adapter‐contaminated reads, and those with >10 ambiguous bases were filtered using Fastp. High‐quality reads were aligned to the reference genome using BWA‐MEM with default parameters. BAM files were sorted with Samtools, and PCR duplicates were marked using MarkDuplicates. Base quality recalibration was performed before variant calling. Germline SNPs and indels were identified using Sentieon's Haplotyper and GVCFTyper modules, while somatic variants were detected via Mutect2. Standard hard filtering criteria (as recommended by GATK Best Practices) were applied. Structural variants (SVs) were detected using Manta, which utilizes split reads, soft‐clipped alignments, insert sizes, and inter‐chromosomal mapping events. CNVs were identified using CNVkit's core‐window algorithm. All variants were annotated with the Variant Effect Predictor (VEP) and categorized by genomic location (e.g., exonic, intronic, splicing, intergenic, and UTR regions).

### Pharmacokinetic Analysis using UPLC‐MS/MS

Pharmacokinetic analysis was conducted using ICR mice (equal numbers of males and females), which were randomly assigned to two groups. Group I received a single oral dose of PEITC dissolved in 1% DMSO (16 mg kg^−1^ body weight), while Group II was administered 20 µL of PEITC‐loaded hydrogel via subcutaneous injection, allowing in situ gel formation at the injection site. The blood samples were withdrawn at defined time points (0.5, 1, 1.5, 2, 2.5, 3, 4, 6, 8, 12, 16, and 24 h). Take 50 µL of serum and add 200 µL of acetonitrile to precipitate the protein. After vortex mixing, place the sample on an ice bath and let it stand for 10–15 min. Centrifuge the sample at 15 000 rpm for 10 min and collect the supernatant. It was determined by the UPLC‐MS/MS method and separated on the Waters ACQUITY UPLC HSS T3 chromatographic column (1.8 µm, 2.1 mm × 100 mm). The parent m/z was 164.0100, and the daughter m/z was 130.1900. The liquid chromatographic conditions were as Table S8 (Supporting Information).

### In Vivo Biodistribution Studies

A biodistribution study was conducted using ICR mice (balanced by sex), randomly assigned to two groups. Group 1 received a single oral administration of PEITC (16 mg kg^−1^) dissolved in 1% DMSO. Group 2 was subcutaneously injected with 20 µL of PEITC‐loaded hydrogel (20 mg mL^−1^), which formed an in situ gel at the injection site. At 2 and 4 h post‐administration, animals were euthanized via CO₂ asphyxiation, and major organs—including the liver, kidneys, heart, lungs, and spleen—were collected. Issues were rinsed with ice‐cold PBS and preserved at −80 °C. Before analysis, samples were thawed, washed thoroughly with cold saline, and blotted dry. A 100 µg portion of each tissue was homogenized in 900 µL of acetonitrile to precipitate proteins. After vortexing, samples were placed in an ice bath for 10–15 min, then centrifuged at 15,000 rpm for 10 min. The supernatant was collected for quantification. Tissue homogenates were further clarified by centrifugation at 15700 × g for 15 min at 4 °C. PEITC concentrations in the supernatants were measured via UPLC‐MS/MS, as detailed in the Pharmacokinetic Analysis section.

### Biochemical Test to Assess Liver and Kidney Function

Hepatotoxicity and nephrotoxicity across treatment groups were evaluated after a 21‐day study period by measuring serum biochemical markers. Levels of alanine aminotransferase (ALT), aspartate aminotransferase (AST), blood urea nitrogen (BUN), and creatinine (CRE) were quantified using commercially available assay kits, following the manufacturer's protocols.

### Histopathological Assessment

At the end of the study, mice were euthanized, and liver, kidney, and subcutaneous tissues were collected from each group. The samples were fixed in 10% neutral‐buffered formalin and processed for hematoxylin and eosin (H&E) staining, which was performed by Wuhan Servicebio Technology Co., Ltd. (Wuhan, China) following standard procedures. Pharmacokinetic, toxicological studies and animal care have been approved by the Ethics Committee for Experimental Animal Research, Zhejiang University of Technology. Animals receive appropriate treatment and are used in a scientifically effective and ethical manner (MGS20250423044).

### Database Analysis

The GEPIA and TGCA database was used to analyze tumor prognosis and the expression of SF3A3 in EC (http://gepia.cancer; https://portal.gdc.cancer.gov/). The UCSC Xena database was used to analyze prognosis (https://xenabrowser.net/)). The drugbank database was used to predict SF3A3 inhibitors (https://go.drugbank.com/).

### Statistical Analysis

Statistical analysis were performed using GraphPad Prism (version 10.1.2). The relevant data were typeset and plotted by Adobe Illustrator. Results are presented as mean ± standard deviation (SD). Animal experiments were conducted independently at least 5 nude mice each group, and cell experiments were repeated at least three times. Differences between two groups were analyzed with independent t‐tests. For more than two groups, one‐way ANOVA followed by Tukey's post‐hoc test was used. Statistical significance was set at *p < *0.05.

## Conflict of Interest

The authors declare no conflict of interest.

## Supporting information



Supporting Information

## Data Availability

The data that support the ﬁndings of this study are available from the corresponding author upon reasonable request. Uncropped images of all immunoblotting and uncropped nucleic acid gel images are shown in supporting information.

## References

[1] E. J. Crosbie , S. J. Kitson , J. N. McAlpine , A. Mukhopadhyay , M. E. Powell , N. Singh , Lancet 2022, 399, 1412. 35397864 10.1016/S0140-6736(22)00323-3

[2] C. A. Hamilton , B. Pothuri , R. C. Arend , F. J. Backes , P. A. Gehrig , P. T. Soliman , J. S. Thompson , R. R. Urban , W. M. Burke , Gynecol. Oncol. 2021, 160, 817. 33516529 10.1016/j.ygyno.2020.12.021

[3] A. van den Heerik , N. Horeweg , S. M. de Boer , T. Bosse , C. L. Creutzberg , Int. J. Gynecol Cancer. 2021, 31, 594. 33082238 10.1136/ijgc-2020-001822 PMC8020082

[4] T. Liu , X. Wang , J. Zhai , Q. Wang , B. Zhang , Cancer Biother. Radiopharm. 2021, 36, 521. 32412793 10.1089/cbr.2019.3278

[5] S. M. Shirvani , A. H. Klopp , A. Likhacheva , A. Jhingran , P. T. Soliman , K. H. Lu , P. J. Eifel , Pract Radiat Oncol. 2013, 3, 21. 10.1016/j.prro.2012.03.013 PMC9258613 24674269

[6] N. C. W. Harvey , E. V. McCloskey , P. J. Mitchell , B. Dawson‐Hughes , D. D. Pierroz , J.‐Y. Reginster , R. Rizzoli , C. Cooper , J. A. Kanis , Osteoporos Int. 2017, 28, 1507. 28175979 10.1007/s00198-016-3894-y PMC5392413

[7] A. Papa , E. Zaccarelli , D. Caruso , P. Vici , P. Benedetti Panici , F. Tomao , Expert Opin Investig Drugs. 2016, 25, 31. 10.1517/13543784.2016.1116517 26560489

[8] H. H. Zhang , X. L. Guo , Cancer Chemother. Pharmacol. 2016, 78, 13. 27118574 10.1007/s00280-016-3037-3

[9] A. Sveen , S. Kilpinen , A. Ruusulehto , R. A. Lothe , R. I. Skotheim , Oncogene 2016, 35, 2413. 26300000 10.1038/onc.2015.318

[10] Y. Zhang , J. Qian , C. Gu , Y. Yang , Signal Transduct Target Ther. 2021, 6, 78. 33623018 10.1038/s41392-021-00486-7 PMC7902610

[11] M. Dewaele , T. Tabaglio , K. Willekens , M. Bezzi , S. X. Teo , D. H. P. Low , C. M. Koh , F. Rambow , M. Fiers , A. Rogiers , E. Radaelli , M. Al‐Haddawi , S. Y. Tan , E. Hermans , F. Amant , H. Yan , M. Lakshmanan , R. C. Koumar , S. T. Lim , F. A. Derheimer , R. M. Campbell , Z. Bonday , V. Tergaonkar , M. Shackleton , C. Blattner , J.‐C. Marine , E. Guccione , J. Clin. Invest. 2016, 126, 68. 26595814 10.1172/JCI82534 PMC4701541

[12] H. Dvinge , E. Kim , O. Abdel‐Wahab , R. K. Bradley , Nat. Rev. Cancer 2016, 16, 413. 27282250 10.1038/nrc.2016.51 PMC5094465

[13] R. Sciarrillo , A. Wojtuszkiewicz , Y.,G. Assaraf , G. Jansen , G. J. L. Kaspers , E. Giovannetti , J. Cloos , Drug Resist. Updates 2020, 53, 100728. 10.1016/j.drup.2020.100728 33070093

[14] S. Alsafadi , A. Houy , A. Battistella , T. Popova , M. Wassef , E. Henry , F. Tirode , A. Constantinou , S. Piperno‐Neumann , S. Roman‐Roman , M. Dutertre , M.‐H. Stern , Nat. Commun. 2016, 7, 10615. 26842708 10.1038/ncomms10615 PMC4743009

[15] B. R. Paolella , W. J. Gibson , L. M. Urbanski , J. A. Alberta , T. I. Zack , P. Bandopadhayay , C. A. Nichols , P. K. Agarwalla , M. S. Brown , R. Lamothe , Y. Yu , P. S. Choi , E. A. Obeng , D. Heckl , G. Wei , B. Wang , A. Tsherniak , F. Vazquez , B. A. Weir , D. E. Root , G. S. Cowley , S. J. Buhrlage , C. D. Stiles , B. L. Ebert , W. C. Hahn , R. Reed , R. Beroukhim , Elife 2017, 6, 23268. 10.7554/eLife.23268 PMC5357138 28177281

[16] C. Sun , Cell. Mol. Life Sci. 2020, 77, 3583. 32140746 10.1007/s00018-020-03493-z PMC7452928

[17] M. Palangat , D. G. Anastasakis , D. L. Fei , K. E. Lindblad , R. Bradley , C. S. Hourigan , M. Hafner , D. R. Larson , Genes Dev. 2019, 33, 482. 30842218 10.1101/gad.319590.118 PMC6499322

[18] D. B. Haack , B. Rudolfs , C. Zhang , D. Lyumkis , N. Toor , Nat. Struct. Mol. Biol. 2023, 31, 179. 38057551 10.1038/s41594-023-01150-0 PMC10968580

[19] D. Chen , H. Zhou , Z. Cai , K. Cai , J. Liu , W. Wang , H. Miao , H. Li , R. Li , X. Li , Y. Chen , H.‐Y. Wang , Z. Wen , J. Exp. Clin. Cancer. Res. 2022, 41, 120. 35365208 10.1186/s13046-022-02299-0 PMC8973551

[20] K.‐L. Liu , Y.‐W. Yin , B.‐S. Lu , Y.‐L. Niu , D.‐D. Wang , B. Shi , H. Zhang , P.‐Y. Guo , Z. Yang , W. Li , Cancer Cell Int. 2022, 22, 109. 35248043 10.1186/s12935-022-02475-4 PMC8897952

[21] M. Ciesla , P. C. T. Ngoc , E. Cordero , Á. S. Martinez , M. Morsing , S. Muthukumar , G. Beneventi , M. Madej , R. Munita , T. Jönsson , K. Lövgren , A. Ebbesson , B. Nodin , I. Hedenfalk , K. Jirström , J. Vallon‐Christersson , G. Honeth , J. Staaf , D. Incarnato , K. Pietras , A. Bosch , C. Bellodi , Mol. Cell 2021, 81, 1453. 33662273 10.1016/j.molcel.2021.01.034

[22] L. Wang , L. Hu , J. Sun , J. Zhao , S. Zhou , L. Liu , W. Yu , Y. Hu , D. Zhou , X. Meng , Z. Yuan , H. Zhang , S. Farrington , M. Timofeeva , K. Ding , J. Little , M. Dunlop , E. Theodoratou , X. Li , npj Precision Oncology. 2025, 9, 124. 40301637 10.1038/s41698-025-00906-9 PMC12041606

[23] Y. Gui , X. Qian , Y. Ding , Q. Chen , F. Ye , Y. Ye , Y. Hou , J. Yu , L. Zhao , Cell Death Dis. 2024, 15, 61. 38233377 10.1038/s41419-024-06451-w PMC10794174

[24] S. Liu , J. Zhang , L. Yin , X. Wang , Y. Zheng , Y. Zhang , J. Gu , L. Yang , J. Yang , P. Zheng , Y. Jiang , L. Shuai , X. Cai , H. Wang , Cell Death Dis. 2020, 11, 412. 32487998 10.1038/s41419-020-2617-7 PMC7265432

[25] K. Milde‐Langosch , Eur. J. Cancer 2005, 41, 2449. 16199154 10.1016/j.ejca.2005.08.008

[26] P. Haloi , S. Chawla , V. B. Konkimalla , Eur. J. Pharm. Sci. 2023, 181, 106367. 36572358 10.1016/j.ejps.2022.106367

[27] R. F. Stanley , O. Abdel‐Wahab , Nat Cancer 2022, 3, 536. 35624337 10.1038/s43018-022-00384-z PMC9551392

[28] Y. Liang , T. Tebaldi , K. Rejeski , P. Joshi , G. Stefani , A. Taylor , Y. Song , R. Vasic , J. Maziarz , K. Balasubramanian , A. Ardasheva , A. Ding , A. Quattrone , S. Halene , Leukemia 2018, 32, 2659. 29858584 10.1038/s41375-018-0152-7 PMC6274620

[29] C. Wang , M. Zheng , S. Wang , X. Nie , Q. Guo , L, Gao , X. Li , Y. Qi , J. Liu , B. lin , Biomed Res. Int. 2019, 2019, 2686875. 31355251 10.1155/2019/2686875 PMC6634061

[30] N. You , C. Liu , Y. Gu , R. Wang , H. Jia , T. Zhang , S. Jiang , J. Shi , M. Chen , M. X. Guan , S. Sun , S. Pei , Z. Liu , N. Shen , Nat. Commun. 2024, 15, 9129. 39443442 10.1038/s41467-024-53088-6 PMC11500173

[31] E. Papaemmanuil , M. Cazzola , J. Boultwood , L. Malcovati , P. Vyas , D. Bowen , A. Pellagatti , J. S. Wainscoat , E. Hellstrom‐Lindberg , C. Gambacorti‐Passerini , A. L. Godfrey , I. Rapado , A. Cvejic , R. Rance , C. McGee , P. Ellis , L. J. Mudie , P. J. Stephens , S. McLaren , C. E. Massie , P. S. Tarpey , I. Varela , S. Nik‐Zainal , H. R. Davies , A. Shlien , D. Jones , K. Raine , J. Hinton , A. P. Butler , J. W. Teague , et al., N. Engl. J. Med. 2011, 365, 1384. 21995386 10.1056/NEJMoa1103283 PMC3322589

[32] X. Y. Shao , J. Dong , H. Zhang , Y. S. Wu , L. Zheng , Front. Mol. Biosci. 2020, 7, 577460. 33344502 10.3389/fmolb.2020.577460 PMC7738478

[33] X. Zhang , X. Zhan , T. Bian , F. Yang , P. Li , Y. Lu , Z. Xing , R. Fan , Q. Cliff Zhang , Y. Shi , Nat. Struct. Mol. Biol. 2024, 31, 835. 38196034 10.1038/s41594-023-01188-0

[34] M. Riedel , M. F. Berthelsen , H. Cai , J. Haldrup , M. Borre , S. R. Paludan , H. Hager , M. H. Vendelbo , E. F. Wagner , L. Bakiri , M. K. Thomsen , Oncogene 2021, 40, 2437. 33674748 10.1038/s41388-021-01724-6 PMC7610543

[35] W. Fan , F. Tian , X. Hong , K. Zhu , Y. Zhan , X. Li , X. Wang , X. Wang , L. Cai , Y. Xing , Adv. Sci. 2024, 11, 2404609. 10.1002/advs.202404609 PMC11653629 39555714

[36] H.‐L. Rabben , Y. Kodama , M. Nakamura , A. M. Bones , T. C. Wang , D. Chen , C.‐M. Zhao , A. Øverby , Front. Pharmacol. 2021, 12, 613458. 33897415 10.3389/fphar.2021.613458 PMC8060630

[37] Y. J. Moon , D. A. Brazeau , M. E. Morris , Evid Based Complement Alternat Med. 2011, 2011, 462525. 20953429 10.1155/2011/462525 PMC2952307

[38] Y. Liu , et al., Quant Imaging Med Surg. 2015, 5, 708. 26682141 10.3978/j.issn.2223-4292.2015.06.01 PMC4671963

[39] M. Y. Chung , T. G. Lim , K. W. Lee , World J. Gastroenterol. 2013, 19, 984. 23467658 10.3748/wjg.v19.i7.984 PMC3582010

[40] Y. Wang , S. Wei , J. Wang , Q. Fang , Q. Chai , Mol. Med. Rep. 2014, 10, 543. 24788892 10.3892/mmr.2014.2167

[41] P. Gupta , S. E. Wright , S. H. Kim , S. K. Srivastava , Biochim. Biophys. Acta 2014, 1846, 405. 25152445 10.1016/j.bbcan.2014.08.003 PMC4260992

[42] K. Sakao , S. Desineni , E. R. Hahm , S. V. Singh , Prostate 2012, 72, 1104. 22161756 10.1002/pros.22457 PMC3310272

[43] X. Peng , T. Wang , B. Dai , Y. Zhu , M. Ji , P. Yang , J. Zhang , W. Liu , Y. Miao , Y. Liu , S. Wang , J. Sun , Adv. Sci. 2024, 12, 2410769. 10.1002/advs.202410769 PMC11714243 39454114

[44] A. T. Sengal , V. Bonazzi , D. Smith , C. P. Moiola , R. Lourie , R. Rogers , E. Colas , A. Gil‐Moreno , S. Frentzas , N. Chetty , L. Perrin , P. M. Pollock , npj Precision Oncology 2023, 7, 127. 38062117 10.1038/s41698-023-00478-6 PMC10703877

[45] S. Yin , Y. Yu , N. Wu , M. Zhuo , Y. Wang , Y. Niu , Y. Ni , F. Hu , C. Ding , H. Liu , X. Cheng , J. Peng , J. Li , Y. He , J. Li , J. Wang , H. Zhang , X. Zhai , B. Liu , Y. Wang , S. Yan , M. Chen , W. Li , J. Peng , F. Peng , R. Xi , B. Ye , L. Jiang , J. J. Xi , Cell Stem Cell. 2024, 31, 717. 38593797 10.1016/j.stem.2024.03.008

[46] S. Dayalan Naidu , T. Suzuki , M. Yamamoto , J. W. Fahey , A. T. Dinkova‐Kostova , Mol. Nutr. Food Res. 2018, 62, 1700908. 29710398 10.1002/mnfr.201700908 PMC6175120

[47] S. Chen , Y. Zhou , Y. Chen , J. Gu , Bioinformatics 2018, 34, i884. 30423086 10.1093/bioinformatics/bty560 PMC6129281

[48] B. Langmead , S. L. Salzberg , Nat. Methods 2012, 9, 357. 22388286 10.1038/nmeth.1923 PMC3322381

[49] F. Ramírez , D. P. Ryan , B. Grüning , V. Bhardwaj , F. Kilpert , A. S. Richter , S. Heyne , F. Dündar , T. Manke , Nucleic Acids Res. 2016, 44, W160. 27079975 10.1093/nar/gkw257 PMC4987876

[50] Y. Zhang , T. Liu , C. A. Meyer , J. Eeckhoute , D. S. Johnson , B. E. Bernstein , C. Nusbaum , R. M. Myers , M. Brown , W. Li , X. S. Liu , Genome Biol. 2008, 9, R137. 18798982 10.1186/gb-2008-9-9-r137 PMC2592715

[51] R. Singh , F. Zhang , Q. Li , Stat. Med. 2022, 41, 1884. 35178743 10.1002/sim.9334 PMC9039958

[52] A. R. Quinlan , I. M. Hall , Bioinformatics 2010, 26, 841. 20110278 10.1093/bioinformatics/btq033 PMC2832824

[53] T. L. Bailey , J. Johnson , C. E. Grant , W. S. Noble , Nucleic Acids Res. 2015, 43, W39. 25953851 10.1093/nar/gkv416 PMC4489269

[54] J. T. Robinson , H. Thorvaldsdóttir , W. Winckler , M. Guttman , E. S. Lander , G. Getz , J. P. Mesirov , Nat. Biotechnol. 2011, 29, 24. 21221095 10.1038/nbt.1754 PMC3346182

