# Supplementary material for: SF3A3 Drives Tumorigenesis in Endometrial Cancer by Enhancing c‐FOS Expression and Represents a Potential Therapeutic Target
Source: Adv Sci (Weinh). 2025 Jul 1;12(36):e04184. doi: 10.1002/advs.202504184 (PMC12463058; doi:10.1002/advs.202504184)
Supplement: Supplementary file 1 — Supporting Information [file ADVS-12-e04184-s001.docx]

Supplementary Materials for

**SF3A3 Drives Tumorigenesis in Endometrial Cancer by Enhancing c-FOS Expression and Represents a Potential Therapeutic Target**

Wei Yu et al.

Corresponding author:

**Lidan Hu,** Department of Nephrology, Children’s Hospital, Zhejiang University School of Medicine, National Clinical Research Center for Child Health, Hangzhou 310052, China; Centre for Computational Biology (CCB), Duke-NUS Medical School, 8 College Road, Singapore.

Tel: 13161938970; E-mail address: hulidan@zju.edu.cn.

**Xiangjun Chen,** Department of Nephrology, Children’s Hospital, Zhejiang University School of Medicine, National Clinical Research Center for Child Health, Hangzhou 310052, China.

Tel/Fax: +8657186971812; E-mail address: chenxiangjun@zju.edu.cn.

**Xue Li,** Department of Big Data in Health Science, School of Public Health and The Second Affiliated Hospital, Zhejiang University School of Medicine, Hangzhou, Zhejiang, China

Tel: 18157140559; E-mail address: xueli157@zju.edu.cn.

This file includes:

Figs. S1 to S8

Tables S1 to S8

Uncropped nucleic acid gel images

Uncropped immunoblotting images

**Supplementary figures and figure captions**


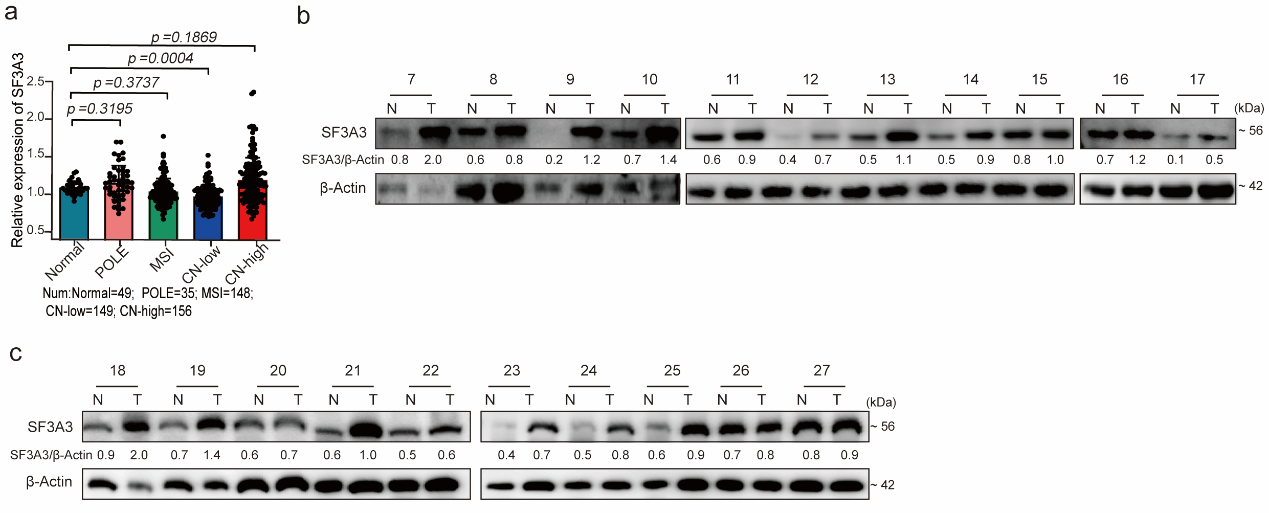


Extended Data Figure 1. SF3A3 is up-regulated in EC and indicates a poor prognosis of EC patients. a), Expression levels of SF3A3 in molecular subtypes of EC, including POLE (ultramutated), MSI (hypermutated), copy-number low (endometrioid), and copy-number high (serous-like) subtypes (https://ngdc.cncb.ac.cn/databasecommons/database/). *p* < 0.05, CN-low group as compared with the Normal group; *p* > 0.05, no significance, CN-high or PLOE or MSI group as compared with the Normal group. b, c), SF3A3 was differentially overexpressed in EC. (*n* = 27 samples each group). Data are presented as mean ± S.D. from at least three independent experiments.


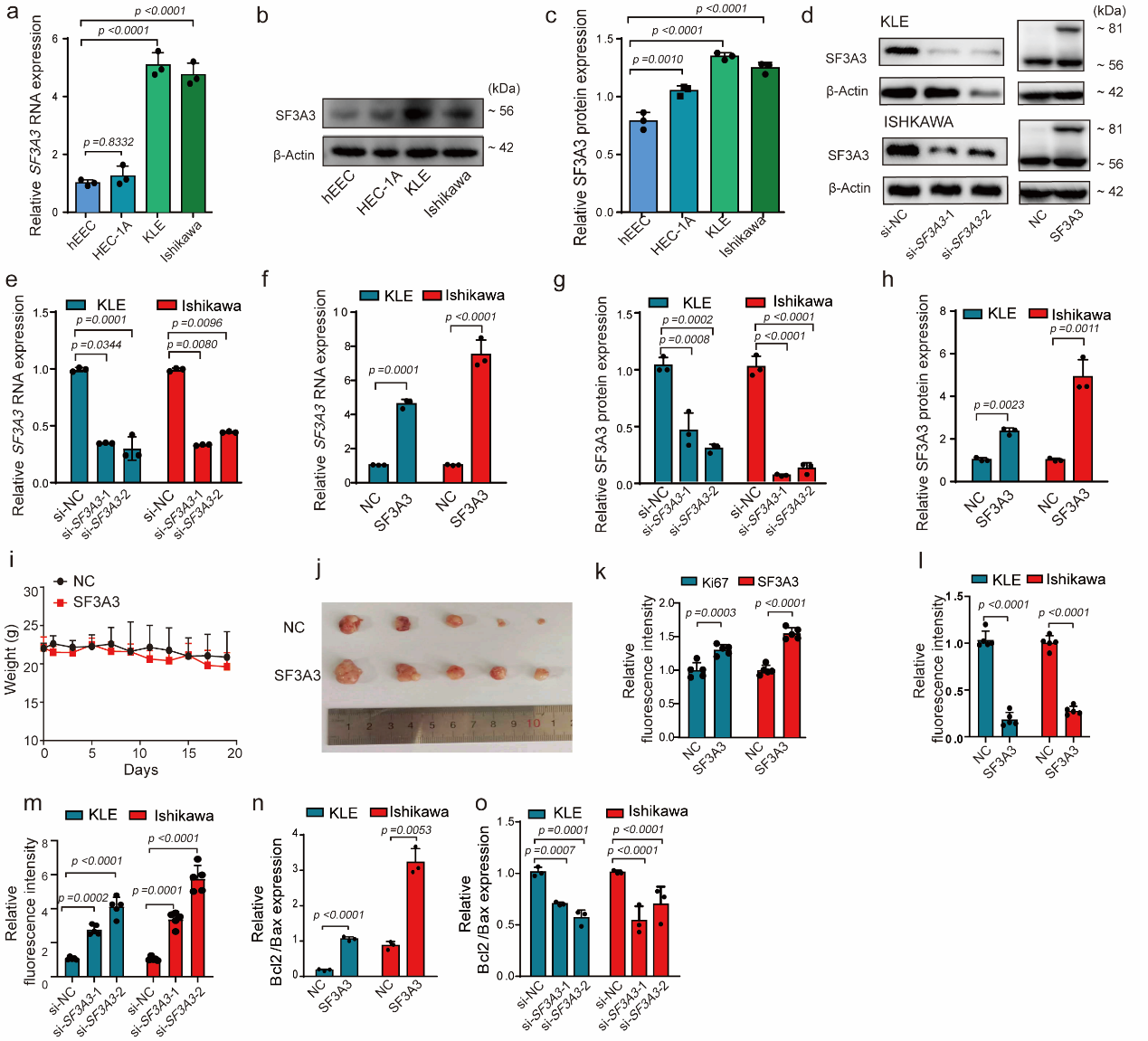
Extended Data Figure 2. Effects of SF3A3 on EC proliferation. a-c), (a)RT-qPCR and (b, c) Immunoblotting analysis of SF3A3 RNA and protein levels in normal endometrial cells hEEC and 3 representative EC cell lines HEC-1A, KLE, Ishikawa. *p* < 0.05, as compared with the hEEC group; (*n* = 3 independent experiments). d), Immunoblotting analysis of SF3A3 expression in EC si-S*F3A3* cells or overexpression cells. (*n* = 3 independent experiments). e, f), RT-qPCR analysis of *SF3A3* expression in EC si-*SF3A3* cells or overexpression cells. *p* < 0.05, as compared with the NC or si-NC group; (*n* = 3 independent experiments). g, h), Immunoblotting analysis of SF3A3 expression in EC si-*SF3A3* cells or overexpression cells. *p* < 0.05, as compared with the NC or si-NC group; (*n* = 3 independent experiments). i), Body weight changes of mice during the experiment. j), Photographs of excised tumor xenografts on day 21. (*n* = 5 samples each group). k), Representative images of SF3A3 and Ki67 immunofluorescence and HE staining in sections of excised tumors. *p* < 0.05, as compared with the NC group; (*n* = 5 samples each group). l, m), Apoptotic cells were detected by TUNEL staining in EC overexpression cells or si-*SF3A3* cells. *p* < 0.05, as compared with the NC or si-NC group; (*n* = 5 samples each group). n, o), Apoptotic markers were measured by immunoblotting in EC si-*SF3A3* cells or overexpression cells. *p* < 0.05, as compared with the NC or si-NC group; (*n* = 3 independent experiments). Scale bars = 50 µm. Data are presented as mean ± S.D. from at least three independent experiments.


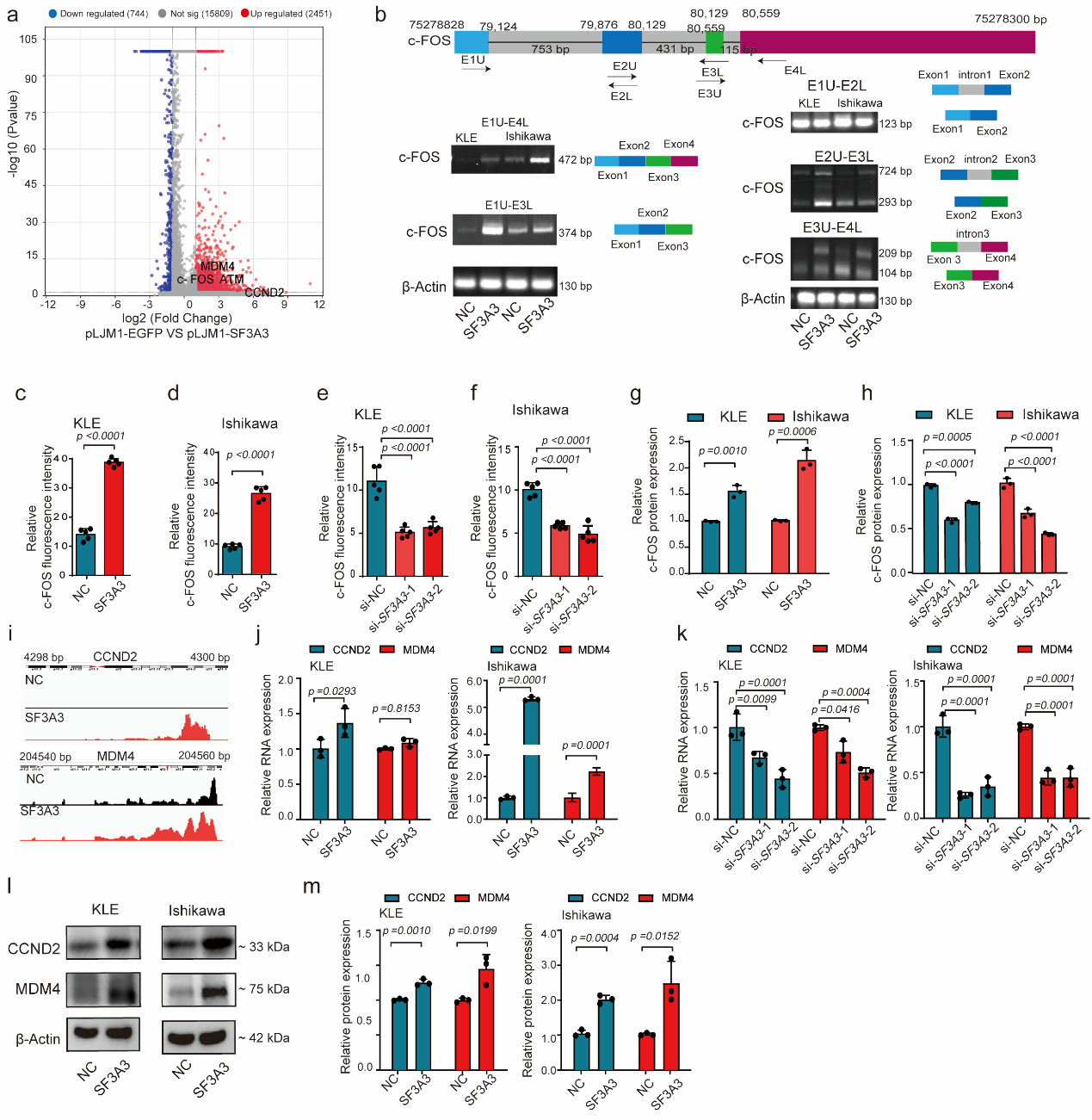
Extended Data Figure 3. Identiﬁcation of the genome-wide SF3A3-binding sites on RNA. a), RIP-seq identified 2,451 genes bound by SF3A3, with significant upregulation of *c-FOS, MDM4,* and other genes. b), RT-PCR assays validating SF3A3-mediated enhancement of alternative splicing in c-FOS. (*n* = 3 independent experiments). c-f), Analysis of FISH experiments showed that overexpression or si-*SF3A3* enhanced or decreases *c-FOS* gene expression. *p* < 0.05, as compared with the NC or si-NC group; (*n* = 3 independent experiments). g, h), Immunoblotting analysis of SF3A3-mediated changes in c-FOS protein levels in KLE and Ishikawa cells. *p* < 0.05, as compared with the NC or si-NC group; (*n* = 3 independent experiments). i), Schematic of the alternative splicing (AS) pattern and SF3A3-binding sites in CCND2 and MDM4. j, k), RT-qPCR analysis of SF3A3-mediated changes in *CCND2* or *MDM4* RNA levels in KLE and Ishikawa cells. *p* < 0.05, as compared with the NC or si-NC group;(*n* = 3 independent experiments). l, m), Immunoblotting analysis of SF3A3-mediated changes in CCND2 or MDM4 protein levels in KLE and Ishikawa cells. *p* < 0.05, as compared with the NC group; (*n* = 3 independent experiments). Data are presented as mean ± S.D. from at least three independent experiments.


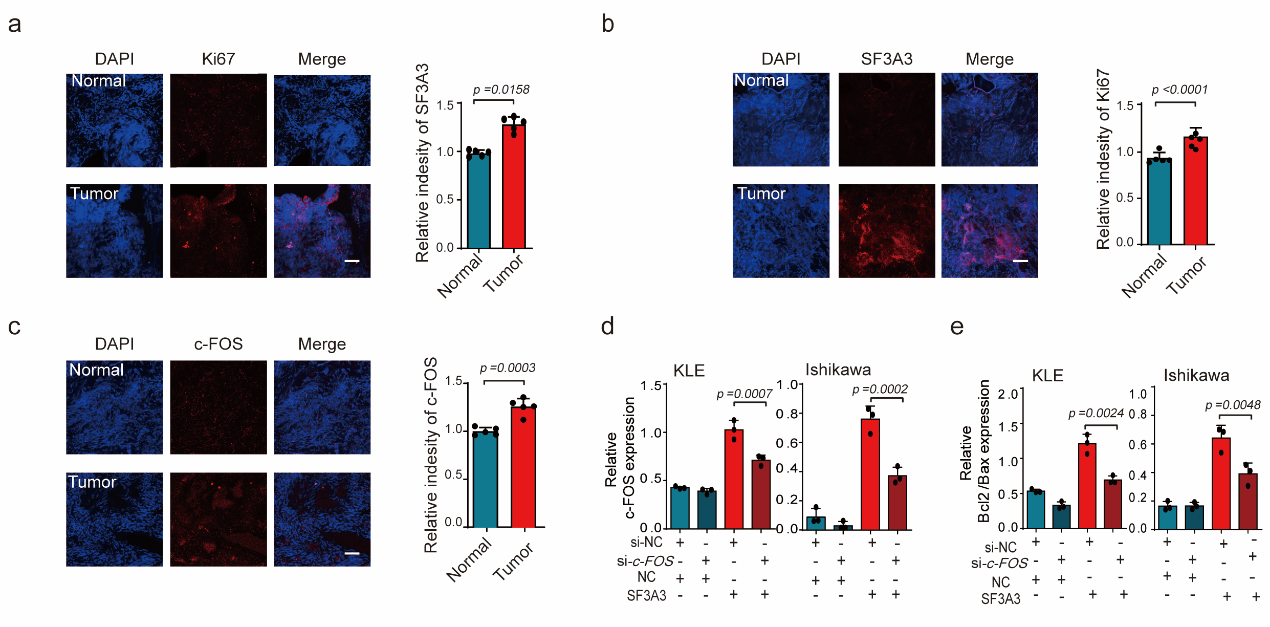
Extended Data Figure 4. The oncogenic roles of SF3A3 in EC are mediated through c-FOS expression. a-c), Representative immunohistochemistry (IHC) images of Ki67, SF3A3 and c-FOS staining in paired adjacent noncancerous endometrium tissues (N) and human EC tissues. Scale bars = 50 µm. *p* < 0.05, as compared with the Normal group; (n = 5 samples each group)**.** d, e), Immunoblotting analysis of c-FOS, Bax, and Bcl2 expression levels in KLE and Ishikawa cells after si-*c-FOS* treatment. *p* < 0.05, SF3A3+siNC group as compared with the SF3A3+si-*c-FOS* group; (*n* = 3 independent experiments). Data are presented as mean ± S.D. from at least three independent experiments.


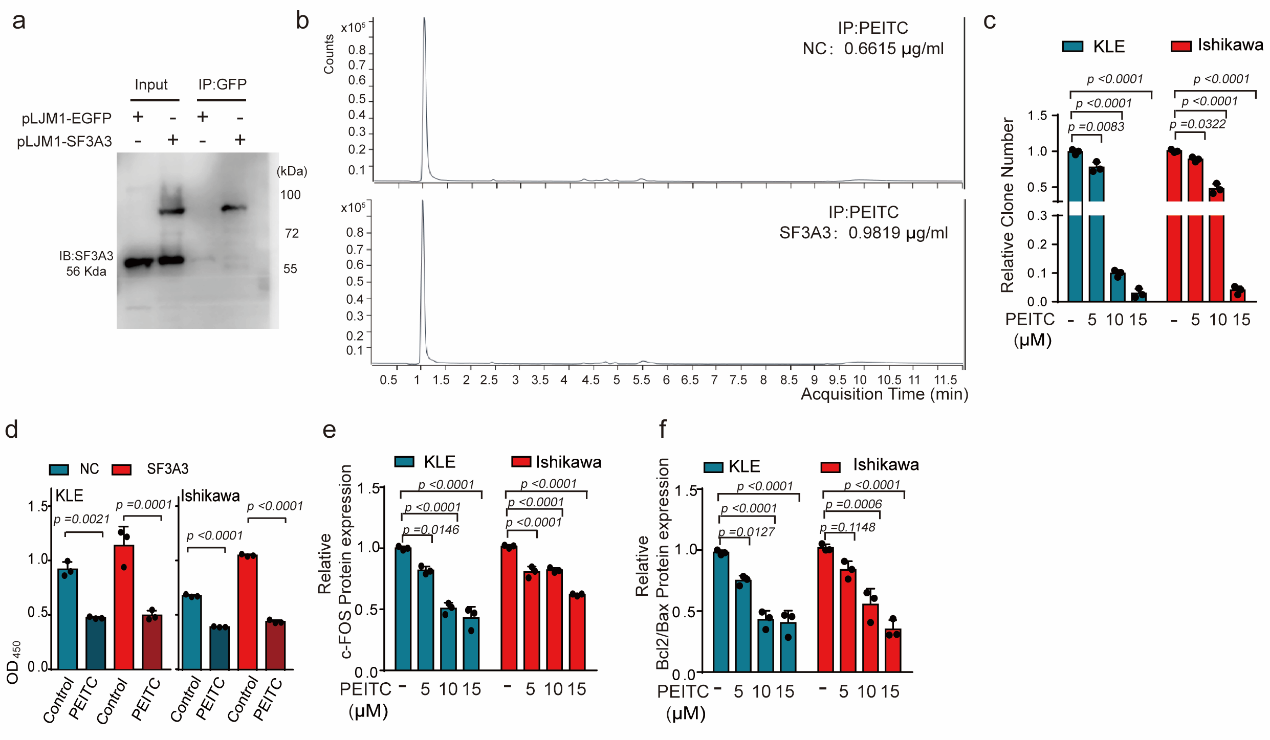
Extended Data Figure 5. PEITC inhibits EC cell proliferation via SF3A3 and induces cell apoptosis. a), Protein validation of SF3A3 IP pulldown. (*n* = 3 independent experiments). b), After IP experiment, NC or SF3A3 group was co-incubated with PEITC and analyzed by mass spectrometry in KLE cells. c), Colony formation by KLE and Ishikawa cells cultured for 10 days with PEITC treatment. Colony formation quantified by light microscopy. *p* < 0.05, as compared with the Control group; (*n* = 3 independent experiments). d), The change of PEITC on EC proliferation with SF3A3 overexpression (PEITC;10 µM). *p* < 0.05, as compared with the Control group; (*n* = 3 independent experiments). e, f), Immunoblotting analysis of c-FOS, Bcl2 and Bax protein levels with PEITC treatment. *p* < 0.05, as compared with the Control group; (*n* = 3 independent experiments). Data are presented as mean ± S.D. from at least three independent experiments.


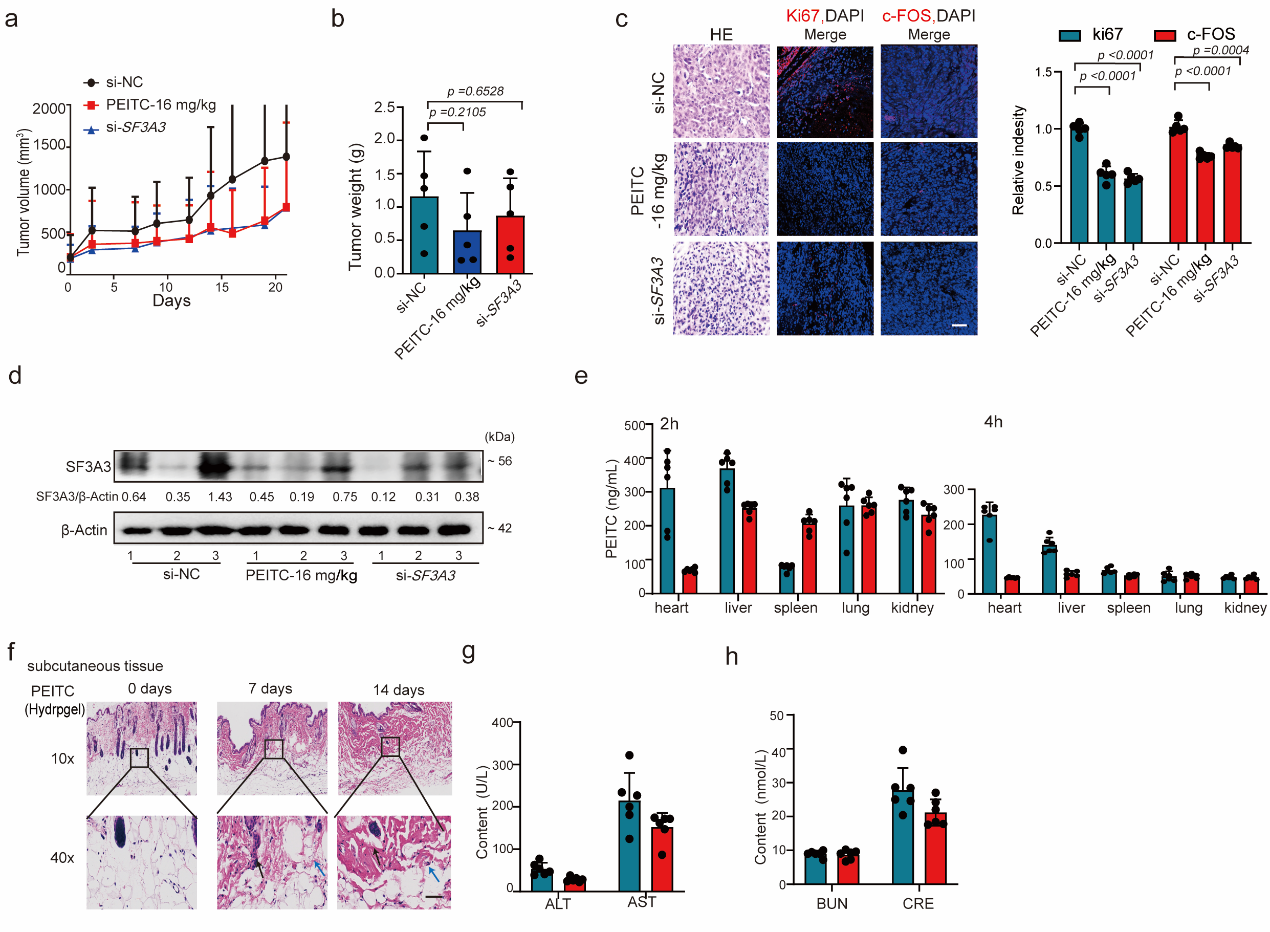
Extended Data Figure 6. Effect of SF3A3 or PEITC on EC xenograft growth in mice. Groups of athymic BALB/c nude mice were injected subcutaneously with 10^6^ KLE cells. After the tumor volume reaches 100mm^3^, 5 nM si-*SF3A3*, si-NC or 16 mg/kg PEITC was injected every 3 days via the intratumoral injection and intragastric administration route. On day 35, animals were sacrificed and tumors samples were collected for analysis. (*n* = 5 samples each group). a), Tumor volumes. b), Tumor weights of tumor xenografts excised on day 35. c), Representative images of c-FOS and Ki67 immunofluorescence and HE staining in sections of excised tumors. Scale bars = 50 µm. *p* < 0.05, as compared with the si-NC group; (*n* = 5 samples each group). d), Immunoblotting analysis of SF3A3 in tumors treated with si-NC, si-*SF3A3* and PEITC treatment (*n* = 3 samples each group). e), *In vivo* biodistribution of free PEITC and PEITC-loaded hydrogels in healthy rats. (*n* = 6 samples each group). Each data point is plotted as mean ± SD from duplicate measurements. f), *In vivo* degradation and inflammatory response were recorded from the images of the excised skin around the implanted site at 0 day, 7 days and 14 days after the injection. Histological images of the subcutaneous tissues (HE stained) surrounding the hydrogels. Scale bars = 20 µm. Black arrow: focal areas of angiotelectasis/dilation of blood vessels, blue arrow: inflammatory cells with necrotic tissue. g, h), Effects of PEITC-loaded hydrogel on hepatic and renal function. (g) hepatic biomarkers (ALT and AST) and (h) renal biomarkers (CRE and BUN). (*n* = 6 samples each group).

**
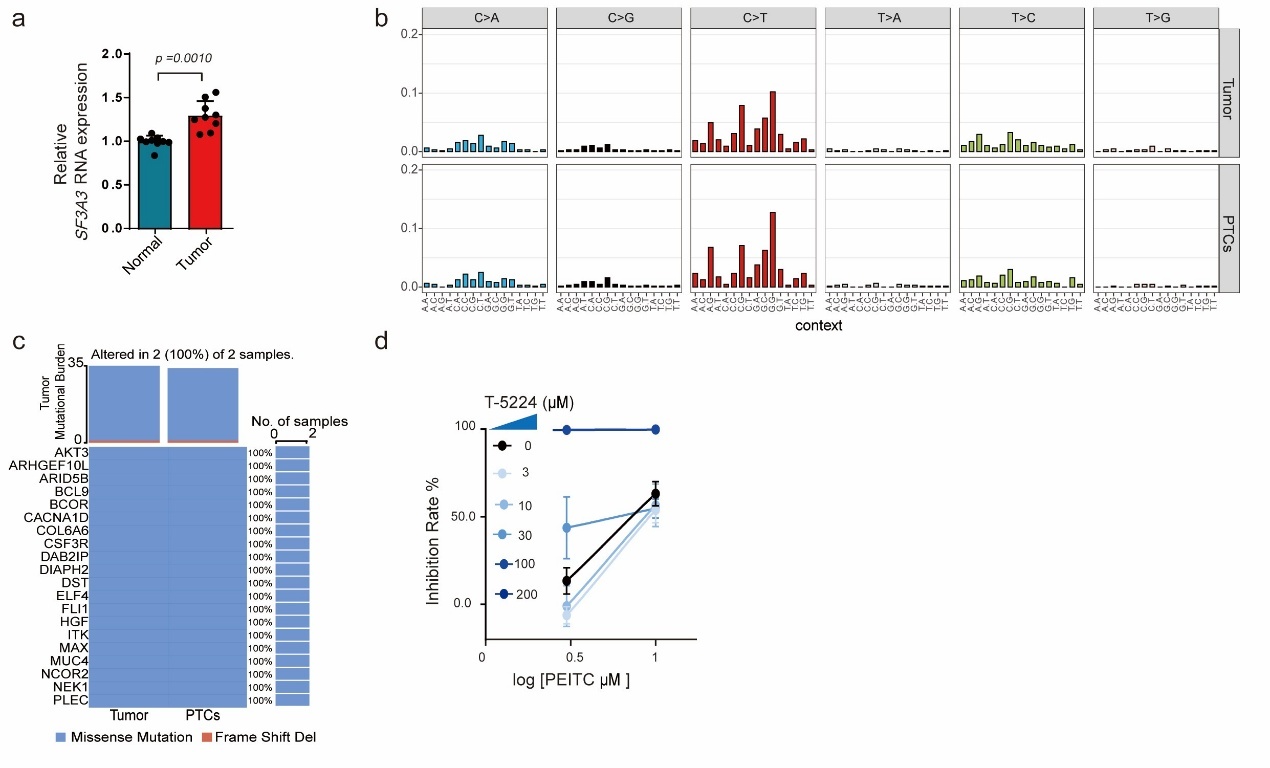
**

Extended Data Figure 7. Effect of PEITC on EC in PTCs. a), RT-qPCR analysis of *SF3A3* in EC tissues. *p* < 0.05, as compared with the Control group; (*n* = 3 samples each group). b), The frequency of 96 types of mutations in tumor samples and PTCs, 6 types of single nucleotide substitutions combined with 16 combinations of bases on both sides, constitute 96 types of trinucleotide substitutions. By using Non negative Matrix Factorization (NMF) method, point mutations are decomposed into multiple different mutation features, and the sample mutation features are clustered with 67 known mutation features (COSMIC-v3.4 SBS GRCH38) on the COSMIC website (http://cancer.sanger.ac.uk/cosmic/signatures). Using annotation information of known features similar to the mutation characteristics of the sample to explain the sample mutation process. c), Somatic mutations identified through whole-exon sequencing (WES) in EC organoids and their parental tumors are summarized. d), Relative cell inhibitions rates plotted as a function of PEITC concentration at differing T-5224 doses.


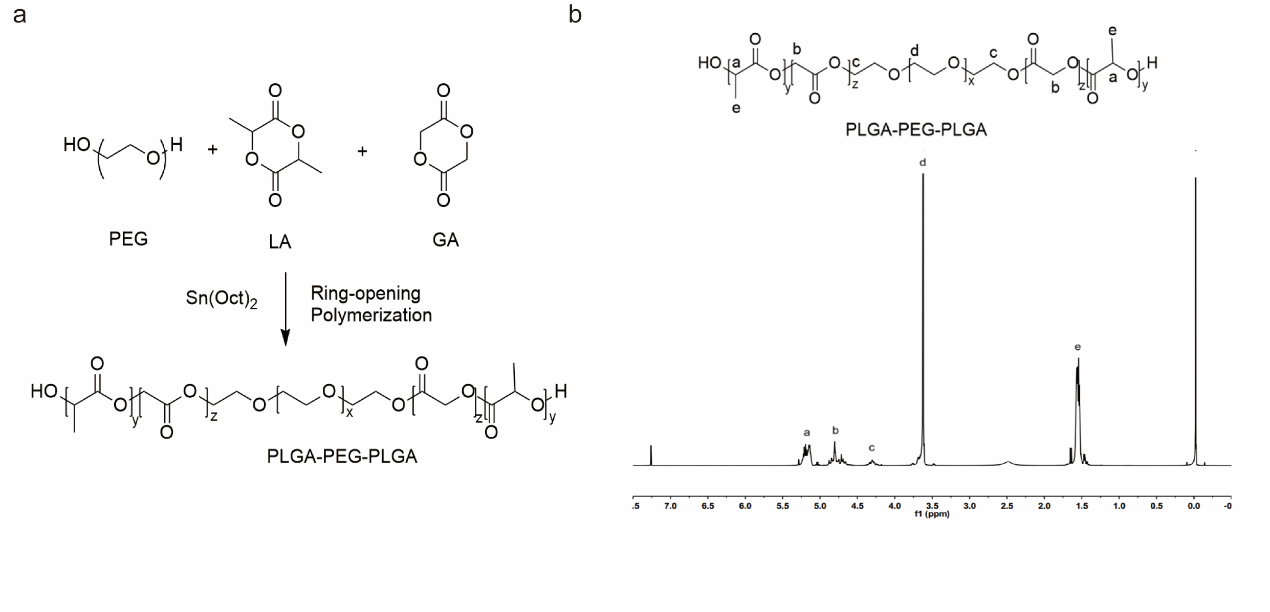
Extended Data Figure 8. PEITC(Hydrogel) synthesis. a), The synthesis route of PLGA-PEG-PLGA. b), The 1H NMR spectra of PLGA-PEG-PLGA.

Table S1. Chemical reagents used in this study

| Chemical reagent | Company | Cat# | Working concentration |
| --- | --- | --- | --- |
| PEITC | Merck | 2257-09-2 | 5-10-15 µM |
| Protease inhibitor cocktail | MCE | HY-K0010 | 1X |
| Phosphatase inhibitor cocktail | MCE | HY-K0023 | 1X |
| Cisplatin | Solarbio | D8810 | 1-3-5 µM |

Table S2. Antibodies used for immunoblotting blotting

| Antibody | Company | Cat# | Host | Dilution |
| --- | --- | --- | --- | --- |
| SF3A3 | Cell Signaling Technology | ab157194 | Rabbit | 1:3000 (WB)  1:200 (IF) |
| Bcl2 | Abclonal | A0208 | Rabbit | 1:3000 (WB) |
| Bax | Proteintech | 50599-2-Ig | Rabbit | 1:3000 (WB) |
| c-FOS | Proteintech | 66590-1-lg | Rabbit | 1:3000 (WB)  1:200 (IF) |
| MDM4 | Proteintech | 28747-1 | Rabbit | 1:3000 (WB) |
| CCND2 | Abclonal | A1773 | Rabbit | 1:3000 (WB) |
| β-Actin | Cell Signaling Technology | #2146 | Rabbit | 1:3000 (WB) |
| GFP | Proteintech | 66002-1-Ig | Mouse | 1:3000 (WB) |
| Ki67 | Abclonal | A20018 | Rabbit | 1:200 (IF) |

Table S3. siRNA targeting sequences

| siRNA | Targeting sequences (5'-3') |
| --- | --- |
| si-*SF3A3*-1 | GGAAGAAUGCAGAGUAUAATT |
| si-*SF3A3*-2 | GCCUAAAUAUCAACUACAATT |
| si-*c-FOS* | CAAGGUGGAACAGUUAUCUTT |

Table S4. Primer sequences for RT-qPCR

| Genes | Forward (5'-3') | Reverse (5'-3') |
| --- | --- | --- |
| *c-FOS* | GCCTCTCTTACTACCACTCACC | AGATGGCAGTGACCGTGGGAAT |
| *MDM4* | GTATCAGAGCAGTTAGGTGTTGG | GACTTAGAGTCCTCCAGGTCATC |
| *SRRM2* | GGCAGCTCTTTTGATCCTCAGC | GTGAGCGAGAACTGCTAGACTC |
| *CCND2* | GAGAAGCTGTCTCTGATCCGCA | CTTCCAGTTGCGATCATCGACG |
| *tnfrsf10b* | TGGAACAACGGGGACAGAACG | GCAGCGCAAGCAGAAAAGGAG |
| *ATM* | TGTTCCAGGACACGAAGGGAGA | CAGGGTTCTCAGCACTATGGGA |
| *PIDD1* | CTTCCAGGTCACACACTTCTCC | AGAGCGATGAGGTTCACACGGT |
| *RAF1* | TCAGGAATGAGGTGGCTGTTCTG | CTCGCACCACTGGGTCACAATT |
| *DIABLO* | GGAGGAAGATGAAGTGTGGCAG | GCCATCTCTGAAAGACCAACTGC |
| *THBS1* | GCTGGAAATGTGGTGCTTGTCC | CTCCATTGTGGTTGAAGCAGGC |
| *TRAF1* | CGATGGCACTTTCCTGTGGAAG | TACAGCCGCAGGCACAACTTGT |
| *ATR* | GGAGATTTCCTGAGCATGTTCGG | GGCTTCTTTACTCCAGACCAATC |
| *MDM2* | TGTTTGGCGTGCCAAGCTTCTC | CACAGATGTACCTGAGTCCGATG |
| *P53* | CCTCAGCATCTTATCCGAGTGG | TGGATGGTGGTACAGTCAGAGC |
| *SF3A3* | CCATGCAAGATATCTGTGTGCC | TCTGTGAACTCCACCAAGTTTTG |
| *β-Actin* | CACCATTGGCAATGAGCGGTTC | AGGTCTTTGCGGATGTCCACGT |

Table S5. Primer sequences for RT-PCR

| Genes | *c-FOS* |  |
| --- | --- | --- |
| E1U | gatagcctctcttactaccactcac | Forward (5'-3') |
| E4L | ctcggtctgcaaagcagacttctca | Reverse (5'-3') |
| E2U | gacctggccgtctccagtgccaacttc | Forward (5'-3') |
| E2L | actgcaggtccggactggtcgagatgg | Forward (5'-3') |
| E3U | agccaaatgccgcaaccggaggag | Forward (5'-3') |
| E3L | atcttattcctttcccttcggattctcc | Reverse (5'-3') |

Table S6. Primer sequences for Hybridization chain reaction (HCR) of *c-FOS*

| Genes | Primer sequences (5’-3’) |
| --- | --- |
| 647 *c-FOS* 1-Forword | CCTCGTAAATCCTCATCAAACTCGTAGTCTGCGTTGAAGCCCGAG |
| 647 *c-FOS* 1-Reverse | CGCGCTGCTGCAGCGGGAGGATGACAAATCATCCAGTAAACCGCC |
| 647 *c-FOS* 2-Forword | CCTCGTAAATCCTCATCAAATAGTAAGAGAGGCTATCCCCGG |
| 647 *c-FOS* 2-Reverse | TGGAGAAGGAGTCTGCGGGTGAGTAAATCATCCAGTAAACCGCC |
| 647 *c-FOS* 3-Forword | CCTCGTAAATCCTCATCAAAAGTCCTGCGCGTTGACAGGCG |
| 647 *c-FOS* 3-Reverse | AGACGGCCAGGTCCGTGCAAAATCATCCAGTAAACCGCC |
| 647 *c-FOS* 4-Forword | CCTCGTAAATCCTCATCAAAGATGGCAGTGACCGTGGGAATGAAG |
| 647 *c-FOS* 4-Reverse | CAGCCACTGCAGGTCCGGACTGGTCAAATCATCCAGTAAACCGCC |
| 647 *c-FOS* 5-Forword | CCTCGTAAATCCTCATCAAAGCGATGGGGCCACGGAGGAGA |
| 647 *c-FOS* 5-Reverse | CTCCGAAAGGGTGAGGGGCTCTGGTAAATCATCCAGTAAACCGCC |
| 647 *c-FOS* 6-Forword | CCTCGTAAATCCTCATCAAACCTGGAGTAAGCCCCAGCGGA |
| 647 *c-FOS* 6-Reverse | TCCTGTCATGGTCTTCACAACGCCAAAATCATCCAGTAAACCGCC |
| 647 *c-FOS* 7-Forword | CCTCGTAAATCCTCATCAAACTTGCCCCTCCTGCCAATGCTCTG |
| 647 *c-FOS* 7-Reverse | TTCTTCTTCTGGAGATAACTGTTCCAAATCATCCAGTAAACCGCC |
| 647 *c-FOS* 8-Forword | CCTCGTAAATCCTCATCAAATATTCCTTTCCCTTCGGATTCTC |
| 647 *c-FOS* 8-Reverse | GTTGCGGCATTTGGCTGCAGCCATAAATCATCCAGTAAACCGCC |
| 647 *c-FOS* 9-Forword | CCTCGTAAATCCTCATCAAATCCGCTTGGAGTGTATCAGTCAG |
| 647 *c-FOS* 9-Reverse | CTTCTCATCTTCTAGTTGGTCTGAAATCATCCAGTAAACCGCC |
| 647 *c-FOS* 10-Forword | CCTCGTAAATCCTCATCAAACTTCAGCAGGTTGGCAATCTCGGTC |
| 647 *c-FOS* 10-Reverse | CAGGATGAACTCTAGTTTTTCCTTCAAATCATCCAGTAAACCGCC |
| 647 *c-FOS* 11-Forword | CCTCGTAAATCCTCATCAAACATCAGGGATCTTGCAGGCAGGTCG |
| 647 *c-FOS* 11-Reverse | CAGACATCTCTTCTGGGAAGCCCAGAAATCATCCAGTAAACCGCC |
| 647 *c-FOS* 12-Forword | CCTCGTAAATCCTCATCAAATGGCAGGCCCCCAGTCAGATCAAG |
| 647 *c-FOS* 12-Reverse | CTCCTCAGACTCCGGGGTGGCAACCAAATCATCCAGTAAACCGCC |
| 647 *c-FOS* 13-Forword | CCTCGTAAATCCTCATCAAAGCTCAGGGTCATTGAGGAGAGGCAG |
| 647 *c-FOS* 13-Reverse | CTTGACAGGTTCCACTGAGGGCTTAAATCATCCAGTAAACCGCC |
| 647 *c-FOS* 14-Forword | CCTCGTAAATCCTCATCAAAGGCTCGGTCTTCAGCTCCATGC |
| 647 *c-FOS* 14-Reverse | TGCTGGGAACAGGAAGTCATCAAAAATCATCCAGTAAACCGCC |
| 647 *c-FOS* 15-Forword | CCTCGTAAATCCTCATCAAAGGGCTGTCTCAGAGCCACTGGG |
| 647 *c-FOS* 15-Reverse | CAGATAGGTCCATGTCTGGCACGGAAAATCATCCAGTAAACCGCC |
| 647 *c-FOS* 16-Forword | CCTCGTAAATCCTCATCAAATCCCAGTCTGCTGCATAGAA |
| 647 *c-FOS* 16-Reverse | CAGGGAGCCACTGTGCAGAGAAATCATCCAGTAAACCGCC |
| 647 *c-FOS* 17-Forword | CCTCGTAAATCCTCATCAAAAGGGGCTCCAGCTCTGTGGCCA |
| 647 *c-FOS* 17-Reverse | GAGTACAGGTGACCACCGGAGTGCAAATCATCCAGTAAACCGCC |
| 647 *c-FOS* 18-Forword | CCTCGTAAATCCTCATCAAAGAAGGAAGACGTGTAAGCAGTGCAG |
| 647 *c-FOS* 18-Reverse | GGAGTCAGCCTCGGGGTAGGTGAAGAAATCATCCAGTAAACCGCC |
| 647 *c-FOS* 19-Forword | CCTCGTAAATCCTCATCAAATGGGCAGCTGCACAGCTG |
| 647 *c-FOS* 19-Reverse | GCTCATTGCTGCTGCTGCCCTTGCAAATCATCCAGTAAACCGCC |
| 647 *c-FOS* 20-Forword | CCTCGTAAATCCTCATCAAAGTGAGCTGAGCGAGTCAGAGG |
| 647 *c-FOS* 20-Reverse | CACAGGGCCAGCAGCGTAAATCATCCAGTAAACCGCC |

Table S7. Clinical patient information

| Patient number | Age | FIGO | differentiation | Pathological type | Lymph nodemetastasis | ER | PR | Ki67 | P53 | MSI | |
| --- | --- | --- | --- | --- | --- | --- | --- | --- | --- | --- | --- |
| 1 | 49 | IA1 | Low-middle | Adenocarcinoma | NO | ++ | ++ | 70% | wild type | MLH1(+)；MSH2(+)  MSH6(+)；PMS2(+) | |
| 2 | 52 | IA1 | High-middle | Adenocarcinoma | NO | 80% | 90% | 20% | 0 | MLH1(+)；MSH2(+)  MSH6(+)；PMS2(weak+) | |
| 3 | 54 | IA2 | Low-middle | Adenocarcinoma | NO | Local + | - | 40% | 60%+ | MLH1(Local+)；MSH2(+)  MSH6(+)；PMS2(Local+) | |
| 4 | 49 | IA2 | Middle | Adenocarcinoma | NO | Local + | Local + | 70% | 70%+ | MLH1(+)；MSH2(+)  MSH6(+)；PMS2(+) | |
| 5 | 57 | IA2 | Low-middle | Adenocarcinoma | NO | Local + | Local + | 50% | Locally weak + | MLH1(+)；MSH2(+)  MSH6(+)；PMS2(+) | |
| 6 | 67 | IA2 | Middle | Adenocarcinoma | NO | Locally weak + | Locally weak + | 40% | Locally weak + | MLH1(+)；MSH2(+)  MSH6(+)；PMS2(+) | |
| 7 | 60 | IA2 | Low | Adenocarcinoma | NO | + | Local + | 40% | - | MLH1(+)；MSH2(+)  MSH6(+)；PMS2(+) | |
| 8 | 56 | IA2 | Middle | Adenocarcinoma | NO | Local + | - | 30% | weak + | MLH1(+)；MSH2(+)  MSH6(+)；PMS2(+) | |
| 9 | 56 | IA2 | Middle | Adenocarcinoma | NO | 60% | 70% | 60% | mistranslated mutant | MLH1(+)；MSH2(+)  MSH6(+)；PMS2(+) | |
| 10 | 43 | IA2 | Middle | Adenocarcinoma | NO | 80%，2+ | 90%，2+ | 40% | wild type | MLH1(+)；MSH2(+)  MSH6(+)；PMS2(+) | |
| 11 | 78 | IA2 | Middle | Adenocarcinoma | NO | 60%，1+ | 80%，1+ | 40% | 1+ | MLH1(+)；MSH2(+)  MSH6(+)；PMS2(+) | |
| 12 | 49 | IA2 | Middle | Adenocarcinoma | NO | + | + | 20% | 15% + | MLH1(+)；MSH2(+)  MSH6(+)；PMS2(+) | |
| 13 | 71 | IA2 | Middle | Adenocarcinoma | NO | Local + | - | 40% | weak + | MLH1(+)；MSH2(+)  MSH6(+)；PMS2(+) | |
| 14 | 69 | IA2 | Middle | Adenocarcinoma | NO | 30% | 90% | 40% | wild type | MLH1(+)；MSH2(+)  MSH6(+)；PMS2(+) | |
| 15 | 53 | IA2 | High-middle | Adenocarcinoma | NO | + | + | 30% | weak + | MLH1(+)；MSH2(+)  MSH6(+)；PMS2(+) | |
| 16 | 59 | IA2 | High | Adenocarcinoma | NO | + | + | 20% | wild type | MLH1(+)；MSH2(+)  MSH6(+)；PMS2(+) | |
| 17 | 44 | IA2 | Middle | Adenocarcinoma | NO | 80% | 90% | 40% | 0 | MLH1(+)；MSH2(+)  MSH6(+)；PMS2(weak+) | |
| 18 | 67 | IB | Low-middle | Adenocarcinoma | NO | Local + | Local + | 60% | wild type | MLH1(+)；MSH2(+)  MSH6(+)；PMS2(+) | |
| 19 | 57 | IIIA1 | Middle | Adenocarcinoma | NO | 25% | 60% | 60% | Local + | MLH1(+)；MSH2(+)  MSH6(+)；PMS2(+) | |
| 20 | 63 | IIIC2 | High | Mixed-type | YES | + | - | 90% | mutant type | MLH1(+)；MSH2(+)  MSH6(+)；PMS2(+) | |
| 21 | 51 | IA2 | Middle | Adenocarcinoma | NO | + | + | 40% | wild type | MLH1(+)；MSH2(+)  MSH6(+)；PMS2(+) | |
| 22 | 51 | IA2 | Middle | Adenocarcinoma | NO | Local + | Local + | 50% | weak+ | MLH1(+)；MSH2(+)  MSH6(+)；PMS2(+) | |
| 23 | 56 | IA2 | / | clear cell carcinoma | NO | - | - | 70% | wild type | MLH1(+)；MSH2(-)  MSH6(-)；;PMS2(+) | |
| 24 | 65 | IA2 | Middle | Adenocarcinoma | NO | + | Local + | 60% | 50%，weak+ | MLH1(+)；MSH2(+)  MSH6(+)；PMS2(-) | |
| 25 | 67 | IB | Low-middle | Adenocarcinoma | NO | Local + | Local + | 60% | wild type | MLH1(+)；MSH2(+)  MSH6(+)；PMS2(+) | |
| 26 | 57 | IIIA1 | Middle | Adenocarcinoma | NO | 25% | 60% | 60% | Local + | MLH1(+)；MSH2(+)  MSH6(+)；PMS2(+) | |
| 27 | 63 | IIIC2 | High | Mixed-type | YES | + | - | 90% | mutant type | | MLH1(+)；MSH2(+)  MSH6(+)；PMS2(+) |

Table S8. liquid chromatographic conditions of UPLC-MS/MS

| Time（min） | 0.1% formic acid-water (%) | Acetonitrile (%) | velocity of flow (mL/min) |
| --- | --- | --- | --- |
| 0.0 | 80 | 20 | 0.2 |
| 4.0 | 5 | 95 | 0.2 |
| 4.5 | 80 | 20 | 0.2 |
| 6.0 | 80 | 20 | 0.2 |


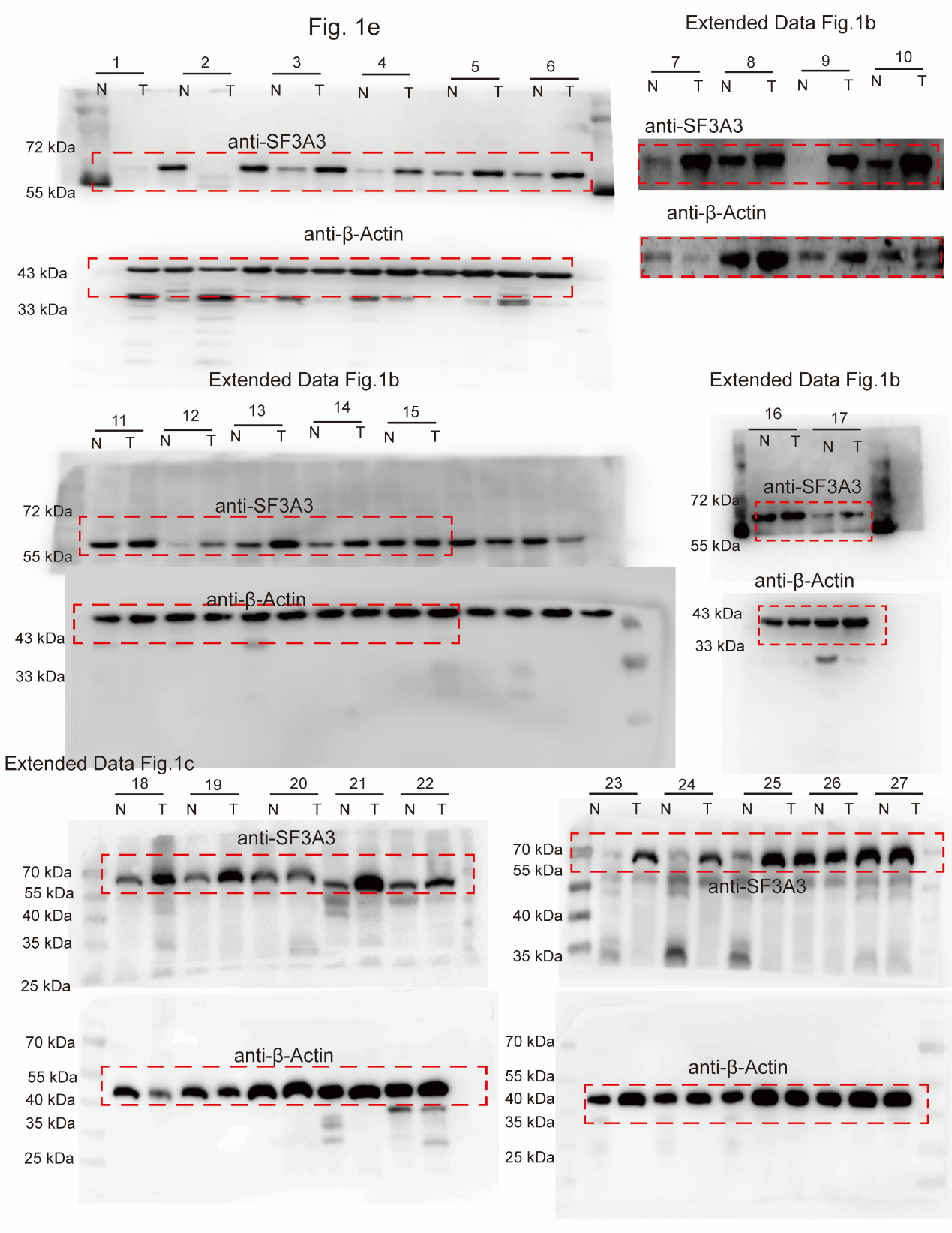
Uncropped immunoblotting images


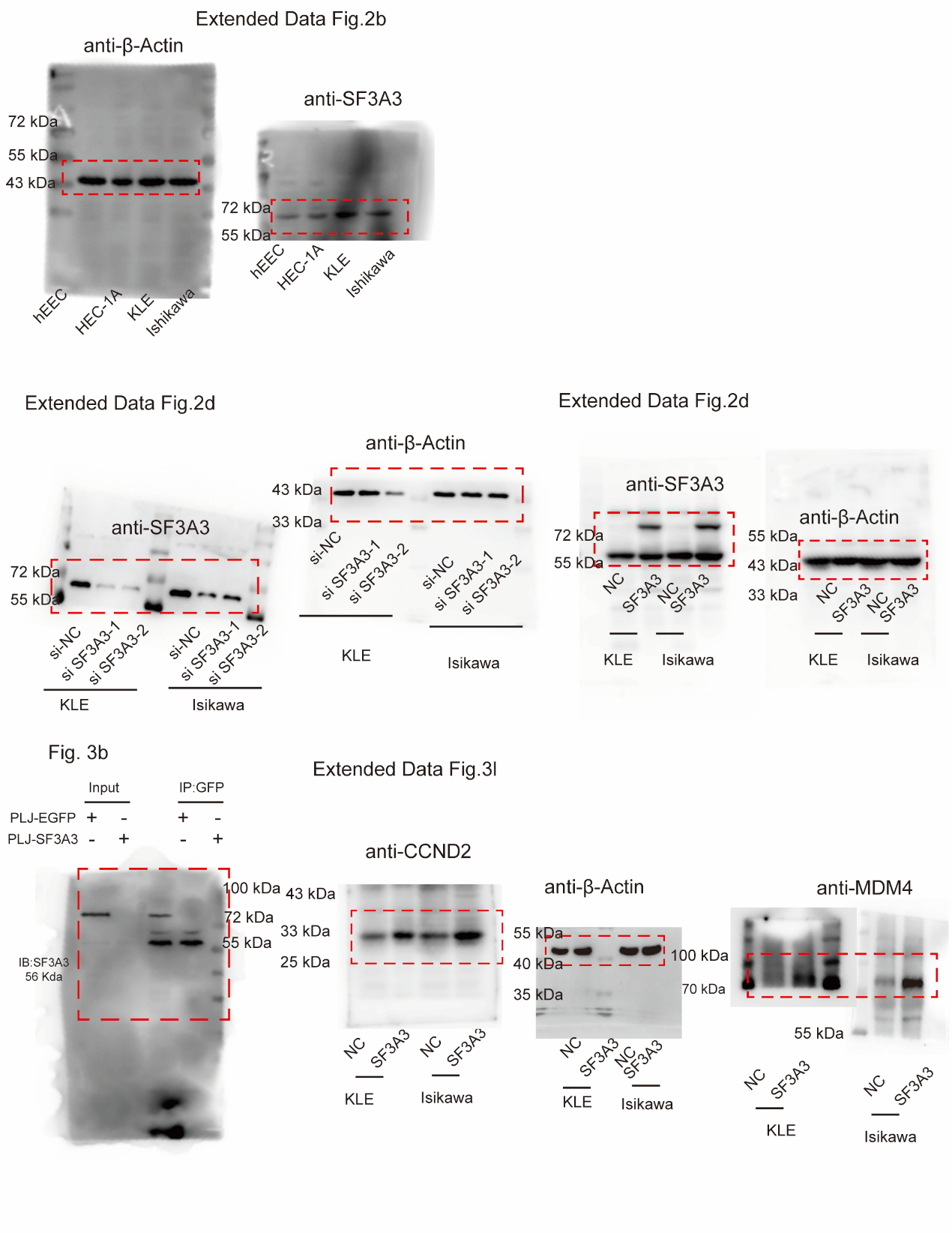


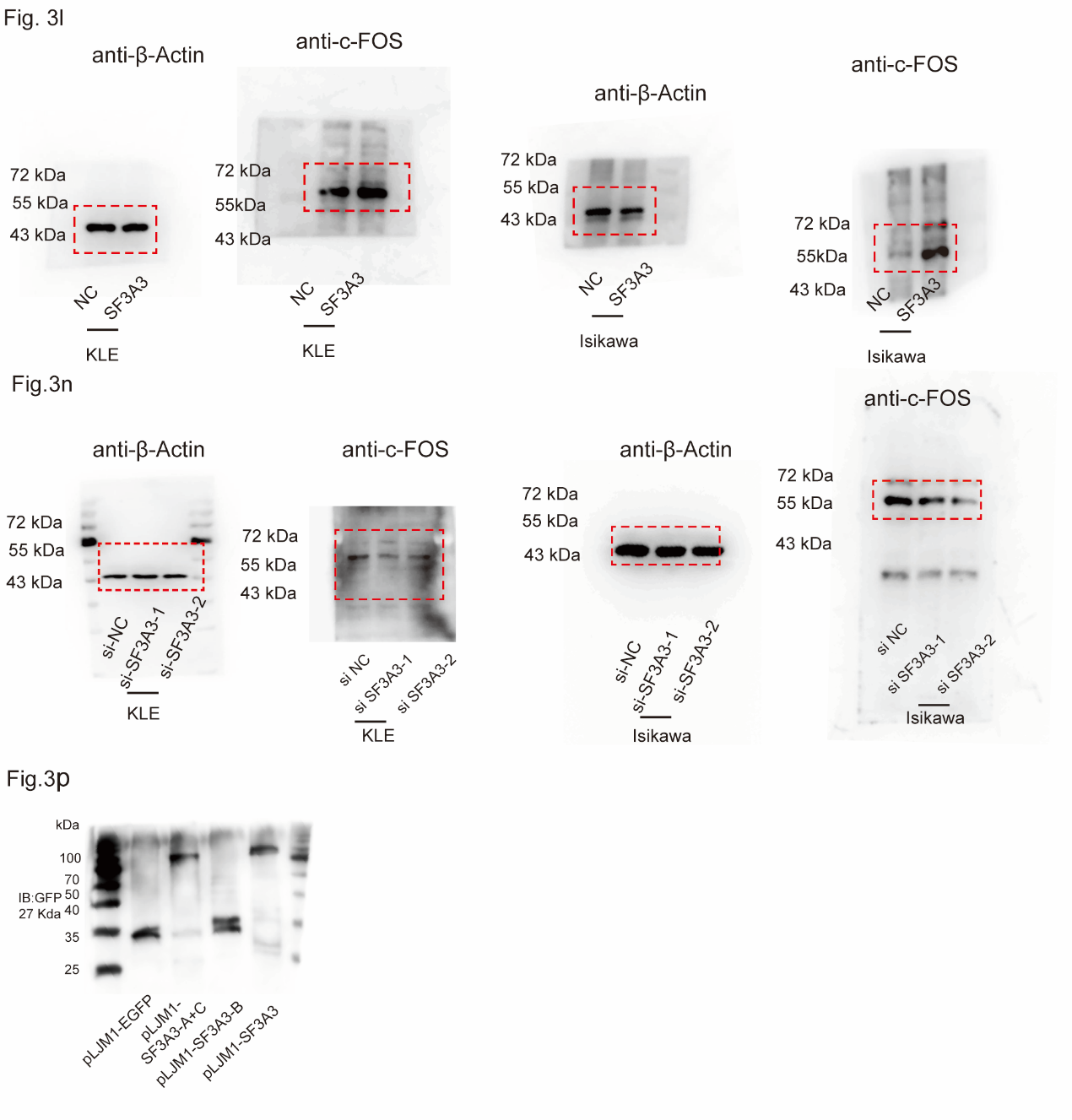


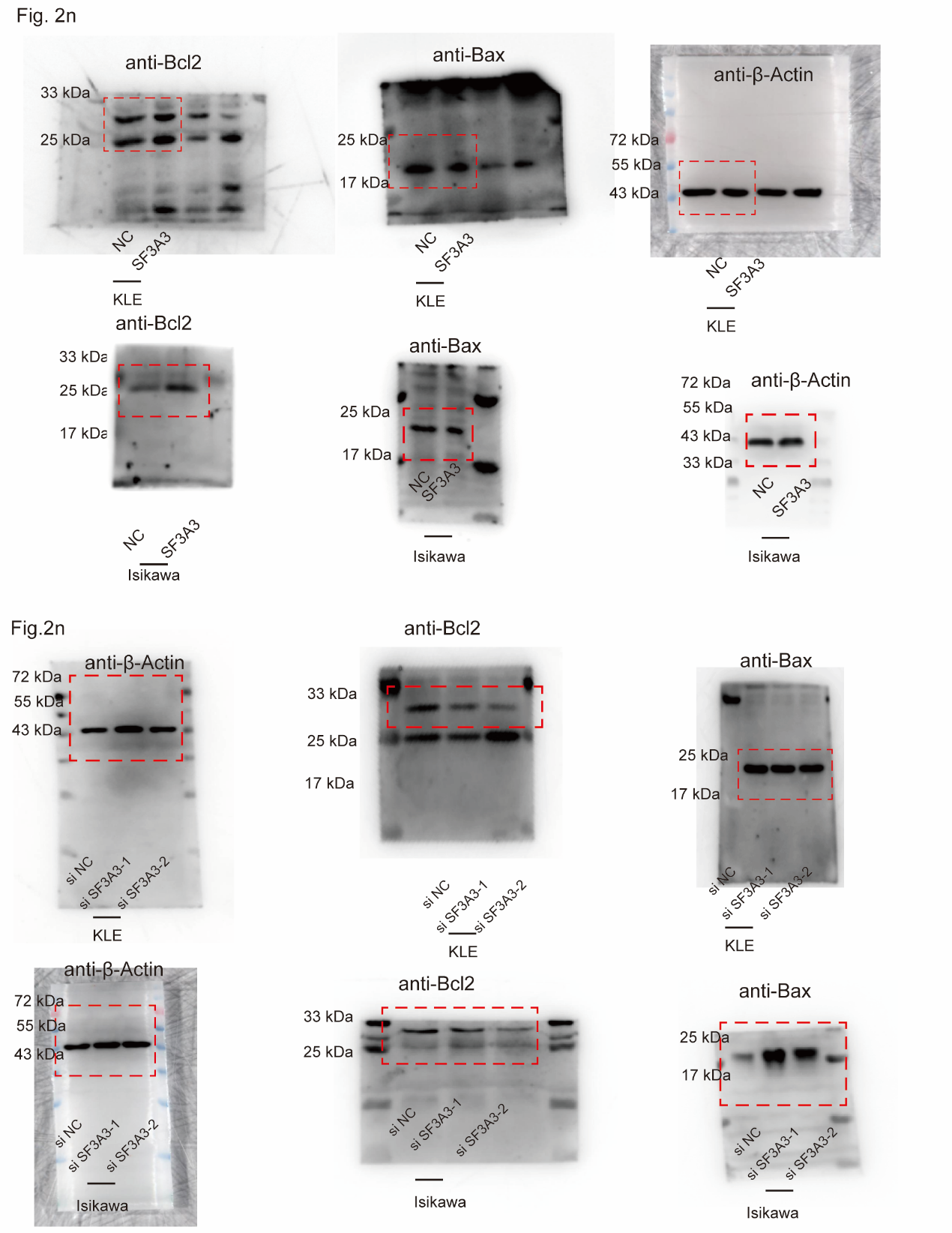


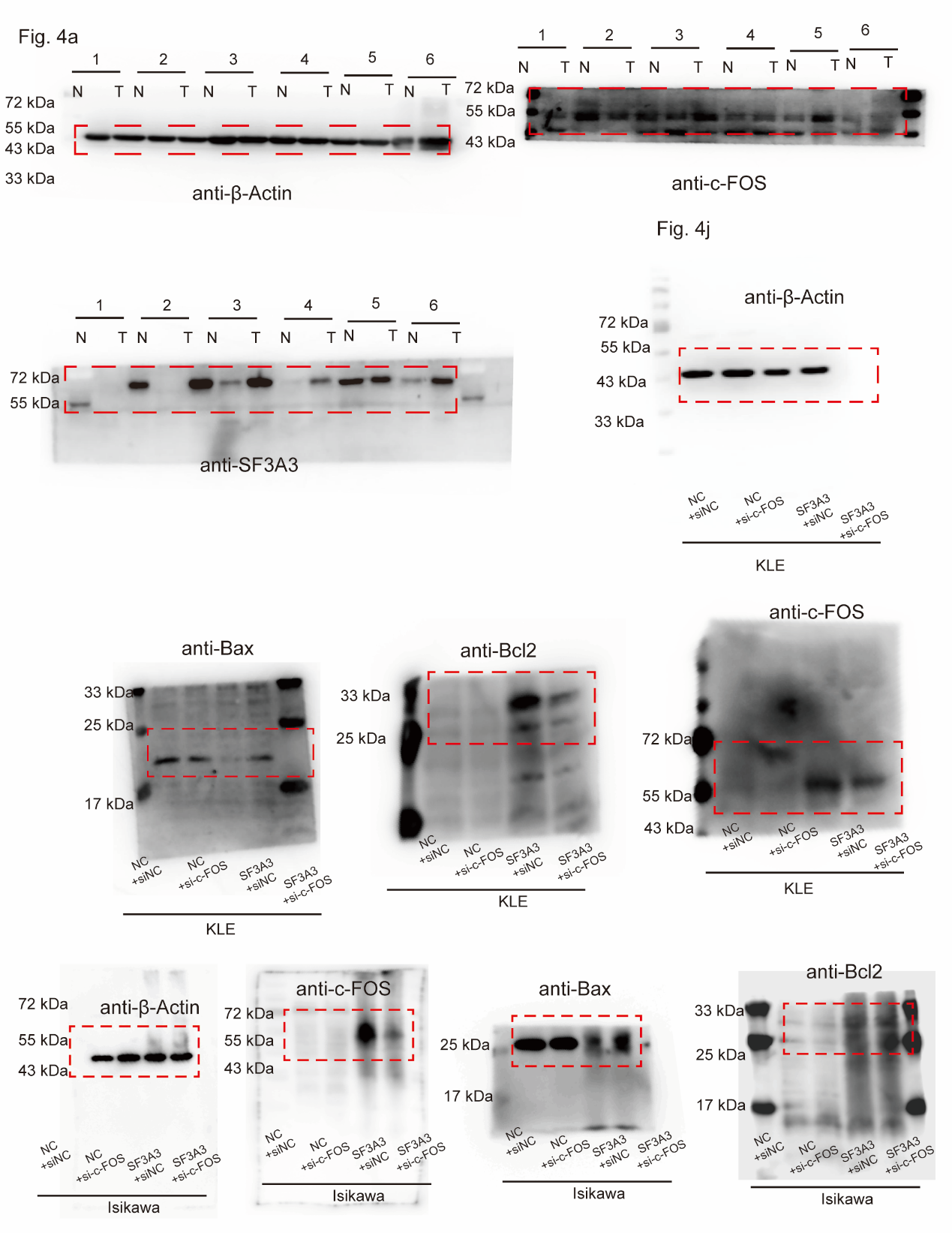


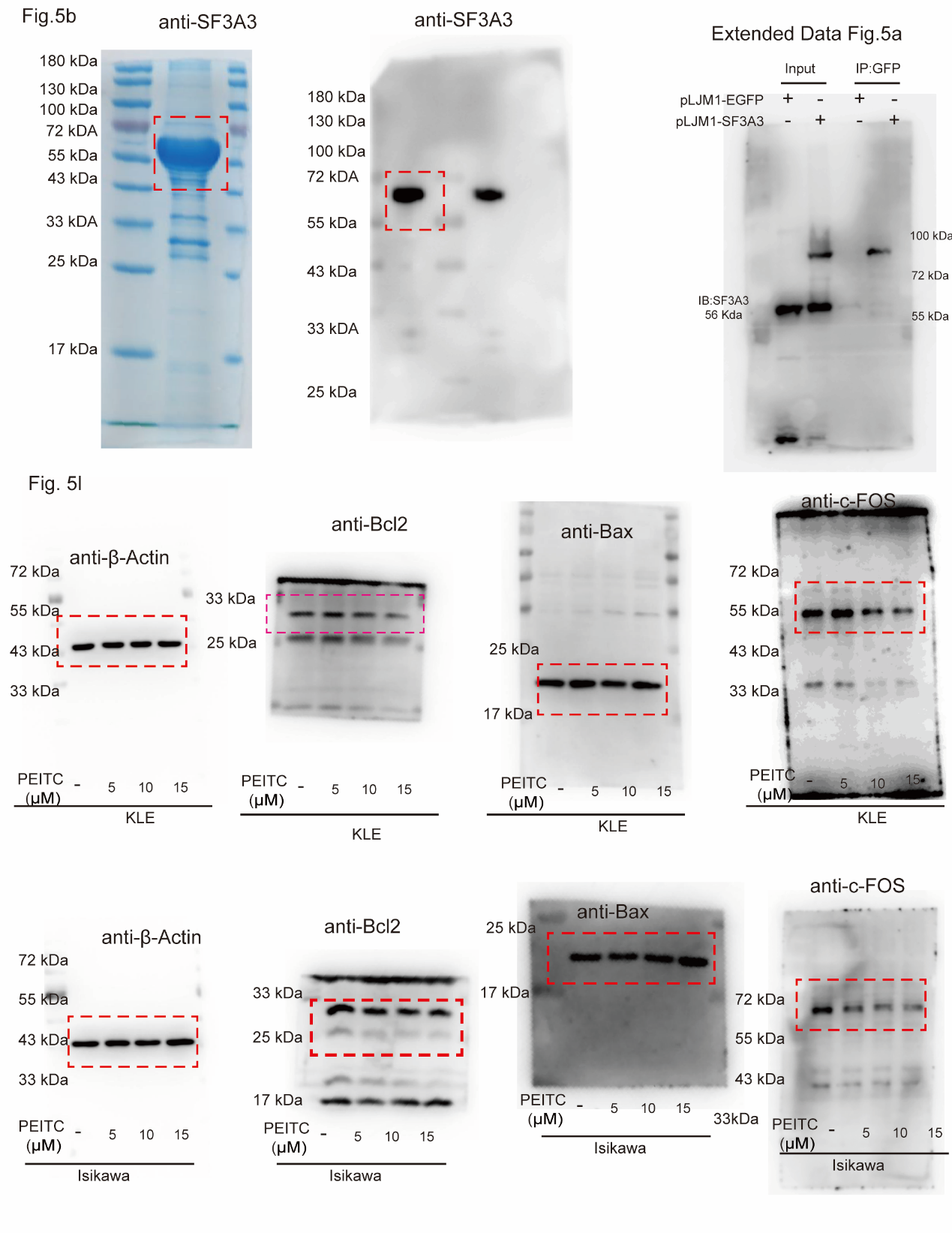


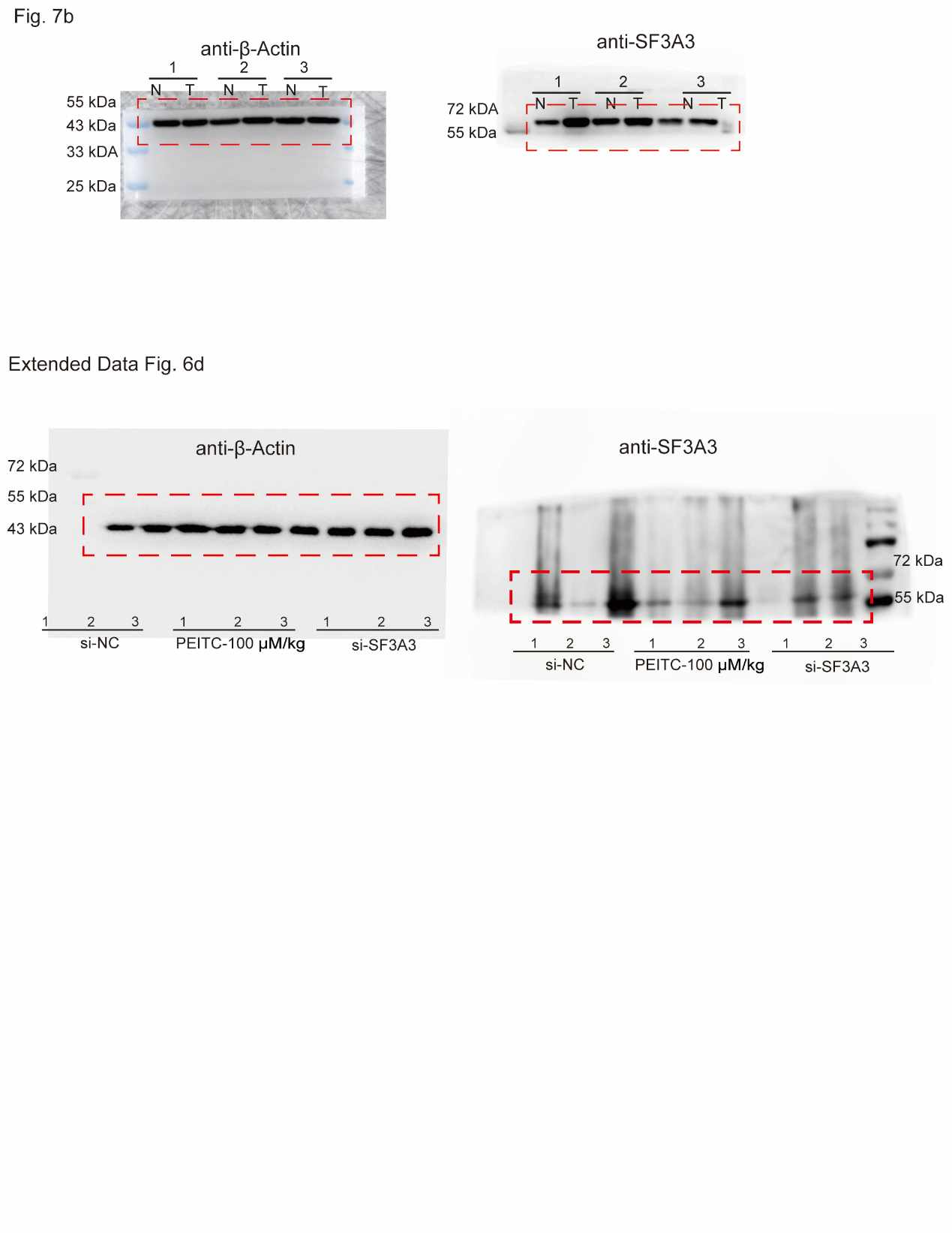


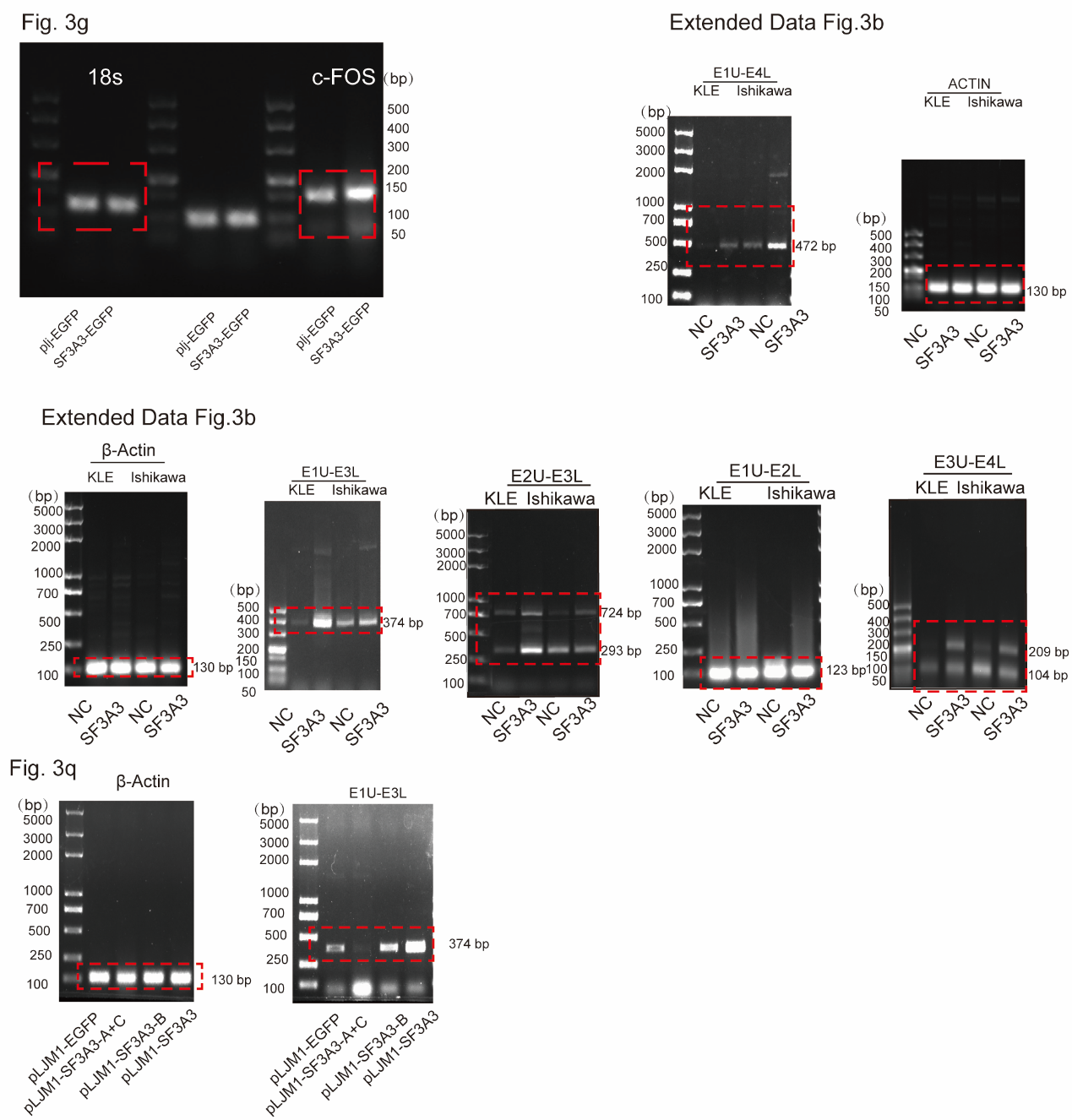
 Uncropped nucleic acid gel
